# Bayesian modeling of human–AI complementarity

**DOI:** 10.1073/pnas.2111547119

**Published:** 2022-03-11

**Authors:** Mark Steyvers, Heliodoro Tejeda, Gavin Kerrigan, Padhraic Smyth

**Affiliations:** ^a^Department of Cognitive Sciences, University of California, Irvine, CA 92697-5100; and; ^b^Department of Computer Science, University of California, Irvine, CA 92697-3435

**Keywords:** human–AI complementarity, Bayesian modeling, image classification, artificial intelligence

## Abstract

Artificial intelligence (AI) and machine learning models are being increasingly deployed in real-world applications. In many of these applications, there is strong motivation to develop hybrid systems in which humans and AI algorithms can work together, leveraging their complementary strengths and weaknesses. We develop a Bayesian framework for combining the predictions and different types of confidence scores from humans and machines. The framework allows us to investigate the factors that influence complementarity, where a hybrid combination of human and machine predictions leads to better performance than combinations of human or machine predictions alone. We apply this framework to a large-scale dataset where humans and a variety of convolutional neural networks perform the same challenging image classification task. We show empirically and theoretically that complementarity can be achieved even if the human and machine classifiers perform at different accuracy levels as long as these accuracy differences fall within a bound determined by the latent correlation between human and machine classifier confidence scores. In addition, we demonstrate that hybrid human–machine performance can be improved by differentiating between the errors that humans and machine classifiers make across different class labels. Finally, our results show that eliciting and including human confidence ratings improve hybrid performance in the Bayesian combination model. Our approach is applicable to a wide variety of classification problems involving human and machine algorithms.

## Significance

With the increase in artificial intelligence in real-world applications, there is interest in building hybrid systems that take both human and machine predictions into account. Previous work has shown the benefits of separately combining the predictions of diverse machine classifiers or groups of people. Using a Bayesian modeling framework, we extend these results by systematically investigating the factors that influence the performance of hybrid combinations of human and machine classifiers while taking into account the unique ways human and algorithmic confidence is expressed.

There has been significant progress over the past decade in the development of machine learning and artificial intelligence (AI) techniques, particularly those based on deep learning methods (1). This has led to new and more accurate methods for addressing problems in areas such as computer vision (2), speech recognition (3), and natural language processing (4). In turn, these techniques are increasingly embedded in commercial real-world applications, ranging from autonomous driving to customer service chatbots (5, 6). While these approaches have produced impressive gains in testbed performance metrics, such as predictive accuracy, it is broadly acknowledged that these approaches have systematic weaknesses and blind spots (7–9). For example, state-of-the-art deep learning classifiers for images and text can fail in surprising and unpredictable ways (10–12).

Thus, hybrid systems where AI algorithms and humans work in partnership are gaining prominence as a focus of both AI and human–computer interaction research (13–17), providing opportunities for more human-centered approaches in the overall design of AI systems (18). An emerging theme in this work is the idea that for many problems, ranging from high risk (medical decisions and autonomous driving) to low risk (automated recommendations on what product or movie to select next), systems that allow humans and AI algorithms to work together are likely to occupy an important part of the spectrum between full autonomy and no autonomy (19–23).

Indeed, there is empirical evidence to suggest that human and machine algorithms working together can be more effective than either working alone, for tasks as varied as face recognition (24), sports prediction (25), diagnostic imaging (26), and classifying astronomical images (27). This prior work demonstrates that humans and machine algorithms can have complementary strengths and weaknesses, possibly resulting from using different sources of information as well as different strategies to process information. For example, in image classification tasks, the differences in processing strategies by humans and machine classifiers lead to different types of errors made by each, even though their overall level of accuracy is similar (28). As a result, a variety of new ideas have emerged on designing crowdsourcing platforms which can leverage algorithmic predictions given limited human resources (29) as well as new theoretical frameworks that optimize machine predictions in the context of working with humans (30–33).

Previous research in decision-making and machine learning has focused on demonstrating the benefits of combining predictions across individuals or algorithms. For example, statistically combining the predictions from a group of individuals often leads to performance better than any individual in the group, especially when the group is diverse (34–38). Similarly, work on ensemble methods in machine learning has shown that combining classifiers is particularly effective when they are less correlated in their predictions (39–42). While much research on human decision-making and machine learning has contributed to our understanding of separate combinations of human (37, 43) or algorithm predictions (44), less is known about the factors that influence hybrid combinations of both.

To systematically investigate these factors, we develop a Bayesian modeling framework that can jointly model human and machine classifier predictions. We apply the framework to a large dataset where humans and a variety of convolutional neural networks (CNNs) perform the same challenging image classification task. CNNs and human visual processing share a number of similarities in terms of their internal representations (45), and the internal representations of CNNs can explain some aspects of human decisions in image classification experiments (46). However, there are also differences in the errors that humans and CNNs make in image classification tasks (28, 47), making image classification an ideal domain to test for complementarity.

With the Bayesian framework, we can empirically and theoretically investigate the conditions that give rise to complementarity. For example, is it better to combine the predictions from a mixture of humans and machine algorithms, leveraging their complementary strengths and weaknesses? Further, when is it better to combine predictions from a group of humans (without algorithms) or a set of machine algorithms (without humans) all performing the same task? Finally, how important is it to differentiate the errors that human and machine algorithms make, and how can we combine qualitatively different expressions of confidence across humans and algorithms?

## Combining Human and Machine Classifier Predictions

The Bayesian combination model we introduce combines the classifications and confidence scores from different ensembles of classifiers, where we use the term “classifier” to refer to either a human or a machine classifier. Although this framework can be applied to any number of classifiers, to simplify the analysis we focus on pairs of classifiers: hybrid human–machine (HM), human–human (HH), and machine–machine (MM) pairs. For each image, the predictions from the two classifiers in the pair are combined leading to a prediction for the pair.

**Fig. 1.**
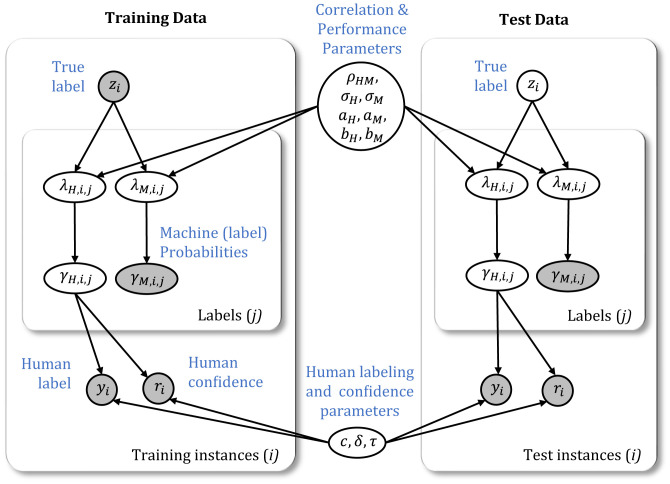
Graphical model of the Bayesian combination model for hybrid HM pairs. Shaded and unshaded nodes represent observed and latent variables, respectively. The plates represent conditionally independent replications of instances (images) and label-related variables per instance.

The modeling approach generates a combined prediction as well as estimates of the latent correlation between classifiers (Fig. 1 and *SI Appendix*, Fig. S1 provide a schematic overview of the generative process assumed by the model). This correlation captures the dependencies across confidence scores of human and/or machine classifications. For example, if one classifier (human or machine) is confident about the label for a particular image, another classifier (human or machine) might show a similar level of confidence about the label for the same image. The correlation between classifiers is a key characteristic of this latent representation and is estimated for the different pair types (HM, HH, and MM). Previous combination models rely on strong conditional independence assumptions (40, 48) or assume that all predictors have the same output types (44, 49, 50) and, hence, fail to address the unique challenges of HM combinations. In particular, previous approaches are not applicable when human and machine classifiers provide different types of confidence scores. For example, machine classifiers (including CNNs) typically produce a probability distribution representing the confidence scores across all labels. In contrast, for the human classifiers it is not practical to request confidence scores for all possible labels. Instead, we model a more typical scenario where a human provides a single confidence score associated with the classification. We assume that human confidence is expressed through a small set of ordinal responses (e.g., “low,” “medium,” and “high”), leading to a different type of confidence score compared to the continuous scores provided by the machine classifier. The difference in confidence scoring between human and machine classifiers is modeled by different generative processes for confidence scoring, operating on the same latent representations.

We first consider the problem of combining the predictions from a hybrid HM classifier pair. We assume there are *N* total images, and for each image a classifier can assign one of *L* possible labels. In addition, both the human and machine classifier are assumed to be noisy labelers relative to the ground truth $z_{i}\in \{1,\ldots ,L\}$ for each image *i*. The generative process starts with a bivariate normal model, conditioned on *z_i_*, to generate latent human and machine classifier logit scores, $\lambda_{H,i,j}$ and $\lambda_{M,i,j}$, separately for each label j∈{1,…,L}, similar to the logit-normal model (51). The ground truth *z_i_* determines which of two bivariate normal distributions is used to generate the logit scores. For each of the labels j∈{1,…,L}, depending on whether *j* agrees with the true label *z_i_* or not, the bivariate distributions have means $\left(\begin{array}{c} a_{H}\\ a_{M} \end{array}\right)$ or $\left(\begin{array}{c} b_{H}\\ b_{M} \end{array}\right)$, respectively:

[1] $$\begin{array}{ll} \left(\begin{array}{l} \lambda_{H,i,j}\\ \lambda_{M,i,j} \end{array}\right) & ∼\{\begin{array}{ll} N((\begin{array}{l} a_{H}\\ a_{M} \end{array}),(\begin{array}{ll} \sigma_{H}^{2} & \sigma_{H}\sigma_{M}\rho_{HM}\\ \sigma_{H}\sigma_{M}\rho_{HM} & \sigma_{M}^{2} \end{array})) & \text{if}\;z_{i}=j\\ N((\begin{array}{l} b_{H}\\ b_{M} \end{array}),(\begin{array}{ll} \sigma_{H}^{2} & \sigma_{H}\sigma_{M}\rho_{HM}\\ \sigma_{H}\sigma_{M}\rho_{HM} & \sigma_{M}^{2} \end{array})) & \text{if}\;z_{i}\neq j \end{array} \end{array}.$$

In this generative model, on a per label basis, the logit scores across classifiers are generated from a multivariate normal distribution which captures the dependencies between labels. The covariance matrix captures the dependencies between logit scores for corresponding labels of the classifiers, where *ρ_HM_* is the (latent) correlation between the two classifiers, and $\sigma^{2}$ is the variance of logit scores. Across the labels, the logit scores for the label that matches the true label have means *a*, and the logit scores for all other labels have means *b*. The difference *a* – *b* determines the ability of the classifier to discriminate between labels. Continuing with the generative process, the logit scores *λ* are then transformed to (normalized) probability confidence scores (i.e., the estimated label probabilities) for both the human and machine classifier:

[2] $$\begin{array}{ll} \gamma_{H,i,j} & \propto \exp(\lambda_{H,i,j})/(1+\exp(\lambda_{H,i,j}))\\ \gamma_{M,i,j} & \propto \exp(\lambda_{M,i,j})/(1+\exp(\lambda_{M,i,j})) \end{array}.$$

For the machine classifier, the *γ_M_* confidence scores are observable for all labels, as produced by the output of the CNN models. For the human classifier, the *γ_H_* confidence scores are latent and assumed to form the basis for generating a single decision and a confidence rating associated with the decision. To produce the human classification *y*, we first apply a softmax rule to the latent confidence scores:

[3] $$\begin{array}{ll} y_{i} & ∼\text{Categorical}\left(\frac{e^{\gamma_{i,1}/\tau }}{\sum_{j}^{L}e^{\gamma_{i,j}/\tau }},\ldots ,\frac{e^{\gamma_{i,L}/\tau }}{\sum_{j}^{L}e^{\gamma_{i,j}/\tau }}\right), \end{array}$$

where we have suppressed the *H* index for readability. The temperature parameter *τ* controls the degree to which the label with the highest probability score determines the classification, modeling the noise that arises in a number of human decision-making contexts (46, 52).

To model the human confidence ratings, we use an ordered probit model that probabilistically maps the latent probability score $\gamma_{i,y_{i}}$ corresponding to the classification made by the human to an ordinal confidence rating, *r_i_*. For our data, we have three confidence ratings (1, low; 2, medium; and 3, high) generated according to

[4] $$\begin{array}{ll} r_{i} & ∼\text{OrderedProbit}(\gamma_{i,y_{i}},c,\delta ), \end{array}$$

where the parameters *c* determine the intervals that map the latent confidence score into a confidence rating and *δ* determines the sharpness of the rating probability curves, i.e., the degree of randomness in the probabilistic mapping from the confidence score to a rating (see *SI Appendix* for details).

The preceding description of the model applies to the case of a hybrid HM pair. For MM pairs, the human is replaced by another machine classifier in Eqs. **1** and **2**, and Eqs. **3** and **4** are left unused. For HH pairs, the machine classifier in Eqs. **1** and **2** is replaced by another human, and Eqs. **3** and **4** are applied separately to each individual human classifier.

### Model Inference.

To apply this model to data, we assume that the ground truth labels (*z*) are observed for a set of training instances and latent for a set of test instances. In addition, human labels, human confidences, and classifier label probabilities are assumed to be observed for both training and test instances. Fig. 1 illustrates the graphical model and inference problem when combining hybrid HM pairs. Conditioned on the observed data for training and test instances, Markov chain Monte Carlo (MCMC) is used to estimate the posterior distribution of the true labels for the test instances, the latent correlation *ρ*, and all other model parameters (*σ*, *a*, *b*, *c*, *δ*, and *τ*) (see *SI Appendix* for details).

For simplicity, the current modeling framework assumes that a single set of parameters (*a_H_*, *b_H_*, *σ_H_*, *c*, *δ*, and *τ*) applies to each individual human classifier as only few observations of the same person are present in the datasets under consideration. However, the framework can be extended to account for individual differences in these parameters.

### Theoretical Limits of Complementarity.

While our Bayesian model allows us to combine human and machine predictions, the general conditions under which complementarity arises are not immediately clear. In this section, we analyze our combination model and derive a condition characterizing complementarity in terms of the accuracies and latent correlations of the classifiers.

Specifically, let *H*_1_ and *H*_2_ be two human classifiers, and let *M*_1_ and *M*_2_ be two machine classifiers. For any pair of classifiers $C_{1},C_{2}\in \{H_{1},H_{2},M_{1},M_{2}\}$, the accuracy of the combined pair of *C*_1_, and *C*_2_ is represented by $A_{C_{1},C_{2}}$. We have complementarity if for some $H\in \{H_{1},H_{2}\}$ and some $M\in \{M_{1},M_{2}\}$, we have $A_{H,M}>\max\left\{A_{H_{1},H_{2}},A_{M_{1},M_{2}}\right\}$. In our analysis, we assume that *H*_1_ and *H*_2_ are exchangeable, as well as *M*_1_ and *M*_2_. Under additional mild assumptions, we derive a necessary and sufficient condition for complementarity in terms of the individual classifier accuracies and correlations (see *SI Appendix* for a detailed proof and discussion of our assumptions).

Our main theoretical result is that the accuracy of the Bayesian combination pair for any unique classifiers *C*_1_ and *C*_2_ can be expressed as

[5] $$A_{C_{1},C_{2}}=\int_{-\infty }^{\infty }\Phi {\left(x\right)}^{L-1}\phi (x-r_{C_{1},C_{2}})\;dx,$$

where Φ(·) represents the cumulative distribution function of a standard Gaussian random variable, and ϕ(·) represents its probability density function. The variable $r_{C_{1},C_{2}}$, which depends on the parameters of our combination model, is defined for each pair type as

[6] $$\begin{array}{ll} r_{H_{1},H_{2}} & =\frac{\left|a_{H}\right|}{\sigma_{H}}\sqrt{\frac{2}{1+\rho_{HH}}}\;\;r_{M_{1},M_{2}}=\frac{\left|a_{M}\right|}{\sigma_{M}}\sqrt{\frac{2}{1+\rho_{MM}}}\\ r_{HM} & =\frac{1}{\sigma \sqrt{1-\rho_{HM}}}\sqrt{\frac{a_{H}^{2}+a_{M}^{2}-2a_{H}a_{M}\rho_{HM}}{1+\rho_{HM}}}. \end{array}$$

Although the integral in Eq. **5** does not have an analytical solution, it can be shown that $A_{C_{1},C_{2}}>A_{C_{1}\prime ,C_{2}\prime }$ if and only if $r_{C_{1},C_{2}}>r_{C_{1}\prime ,C_{2}\prime }$. Hence, complementarity is equivalent to the condition $r_{H,M}>\max\{r_{H_{1},H_{2}},r_{M_{1},M_{2}}\}$. In *SI Appendix*, we further analyze the condition $r_{HM}>\max\{r_{HH},r_{MM}\}$, allowing us to predict complementarity from given model parameters.

Note that according to Eq. **6**, increasing the nonhybrid correlations (*ρ_MM_* and *ρ_HH_*) will always cause the nonhybrid pair accuracies to decrease, thus making complementarity easier to achieve. Similarly, increasing *r_HM_* will increase the hybrid accuracy. However, since *r_HM_* has a more complex dependence on *ρ_HM_*, increasing the hybrid correlation *ρ_HM_* will cause *A_HM_* to decrease if and only if $\min\left(\frac{a_{M}}{a_{H}},\frac{a_{H}}{a_{M}}\right)>\rho_{HM}$. Intuitively, the ratios $a_{M}/a_{H}$ and $a_{H}/a_{M}$ control the relative human–model performance, and higher human–model correlations can be beneficial if the humans and models have vastly different levels of performance.

## Results

To empirically verify our theoretical results and to further investigate the factors that influence complementarity, we collected a large dataset of human and machine classification decisions for a set of 4,800 images. To create variability in machine classifier performance, we selected a number of well-known benchmark CNN architectures (1) for image classification, representative of the recent state of the art in machine classification performance.

To examine conditions for complementarity, we created a number of experimental conditions that lead to variability in performance for human and machine classifiers. One such manipulation is based on adding varying degrees of image noise (47), affecting both human and machine classifier performance. In addition, the classifiers were tuned to the image noise to varying degrees in order to create additional variations in machine classifier performance (*SI Appendix*, Fig. S4).

### Human and Machine Classifiers Make Different Types of Errors.

**Fig. 2.**
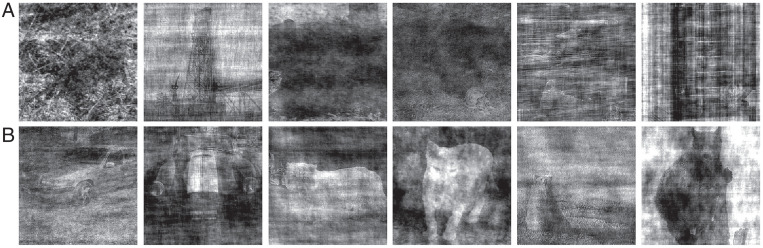
Examples of human and machine classifier complementarity. (*A*) Examples of images that are challenging for humans but relatively easy for machine classifiers. Correct answers in reading order are bird, boat, bear, bear, oven, and oven. (*B*) Examples of images that are challenging for machine classifiers but relatively easy for humans. Correct answers in reading order are car, car, cat, cat, bear, and bear. The machine classifiers in both examples were tuned for one epoch on the noisy images.

Even at comparable levels of performance, human participants and machine classifiers make different types of errors. Fig. 2 shows examples of HM algorithm complementarity. The images in Fig. 2*A* are challenging for humans but relatively easy for machine classifiers. For all of these images, human accuracy and confidence were low (all six human participants made a low-confidence classification, and at most, one out of six human participants made a correct judgment), but machine accuracy was high (at least four out of five machine classifiers made a correct classification for any of these images). The images in Fig. 2*B* are challenging for machine classifiers but relatively easy for humans. All six human judges made a correct and high-confidence classification, whereas at most, one out of five machine classifiers made a correct classification for each of these images.

### Hybrid Combinations of Human and Machine Classifiers Lead to High Accuracy.

**Fig. 3.**
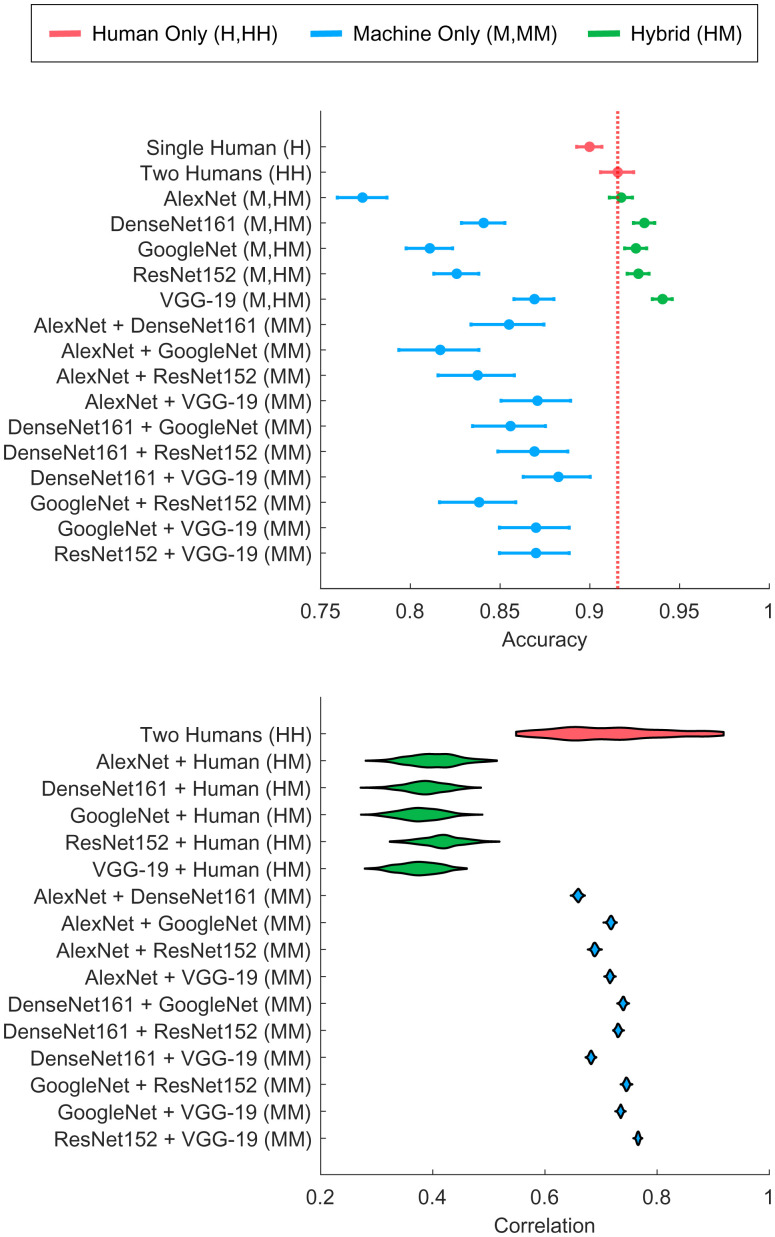
(*Top*) Accuracy results and (*Bottom*) posterior distributions over correlations from the Bayesian combination model. Results are broken down by type of classifiers: single human (H), HH, HM, and MM. Error bars in *Top* reflect 95% confidence interval of the mean based on a binomial model.

For the Bayesian combination model, we created a number of datasets based on three different types of pairs: HH, HM, and MM classifier pairs. As described in *Model Inference*, we use MCMC for inference, with the inferred latent ground truth *z* label and correlation *ρ* being of particular interest. Fig. 3 shows the out-of-sample accuracy results, based on fourfold cross-validation, of the Bayesian combination model. The results are based on low levels of image noise (Ω = 80) and with CNNs that are fine-tuned for one epoch (see *SI Appendix*, Figs. S9–S11 for the results based on other levels of image noise and fine tuning).

Our first finding is that the hybrid pairs of human and machine classifiers perform at a high accuracy relative to nonhybrid combinations such as two humans or two machine classifiers, especially for high levels of image noise. However, for CNNs such as Alexnet (*SI Appendix*), the hybrid combination of Alexnet and a human classifier does not always exceed the performance of a combination of two humans. For this combination, the low baseline performance of the Alexnet classifier does not produce complementarity. The results also show that a combination of two humans leads to better performance than a single human, demonstrating the utility of the human confidence scores—when two human observers differ in confidence, the Bayesian combination model infers that the higher-confidence classification is more likely to be correct.

### Hybrid Combinations of Human and Machine Classifiers Lead to Low Latent Correlations.

Our second finding is that human and machine classifiers produce lower latent correlations than humans do with each other, or than machine classifiers do with each other, demonstrating the utility of combining human and machine predictions—the predictions of hybrid combinations of HM classifiers are more independent than the predictions among humans and machine classifiers alone. Fig. 3, *Bottom*, shows the mean posterior correlations (*ρ*) between classifier combinations. The hybrid HM pairs are correlated less (posterior mean around 0.4) than human-only (posterior mean around 0.7) or machine-only pairs (posterior means between 0.65 and 0.75). Note that the posterior distributions for the machine classifier correlations are associated with low uncertainty due to the availability of a full set of confidence scores across labels for the machine classifiers. In contrast, for the human classifier, only a single confidence rating is available, providing less information to estimate the latent correlational structure.

The inferred pattern of correlation does not critically depend on the representation of the confidence scores. Having only a single continuous confidence score (associated with the classification) and discretizing the machine confidence scores into a small set of ordinal categories, analogous to the human confidence scores, do not change the qualitative pattern of results (*SI Appendix*). This illustrates that the results are robust to different approaches for assessing confidence.

### Accuracy Difference between Classifiers Affects Complementarity.

**Fig. 4.**
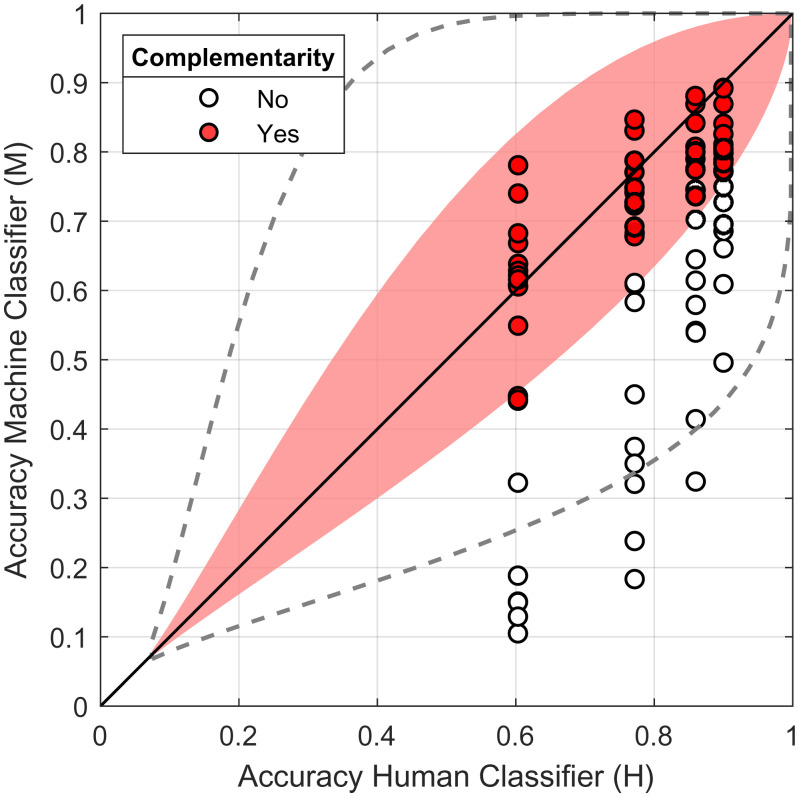
Observed and predicted complementarity as a function of human and machine classifier accuracy. Circles indicate observed accuracy across different datasets, where filled circles indicate combinations where out-of-sample accuracy of the hybrid HM pair outperforms pairs of HH and MM pairs. The colored area shows the area of complementarity as predicted by theory based on $\rho_{HM}=0.33,\;\rho_{HH}=0.62$, and $\rho_{MM\prime }=0.71$, approximately matching the correlations inferred by the Bayesian combination model. The dashed line shows the predicted area of complementarity for a best-case situation where the latent human and model predictions are uncorrelated, $\rho_{HM}=0$, and the nonhybrid correlations remain the same ($\rho_{HH}=0.62,\;\rho_{MM\prime }=0.71$). The diagonal line indicates points of equivalent single human and model performance.

In our third result, we show empirically how accuracy differences between classifiers lead to complementarity and compare the results with theoretical predictions. Fig. 4 shows the observed and predicted complementarity results for a number of hybrid pairs, where the pairs vary in terms of the individual accuracy of the human and machine classifiers composing the pair. Each individual point in the graph is based on the performance of individual classifiers *H* and *M* as well as classifier pairs *HH*, *HM*, and MM′, where *M* and M′ are two different types of CNN classifiers. Complementarity is observed if the hybrid combination *HM* outperforms the combinations consisting of human or machine classifiers alone: $A_{H,M}>A_{H,H}$ and $A_{H,M}>A_{M,M\prime }$. To understand how complementarity varies as a function of the difference between human and machine classifier performance, Fig. 4 shows the out-of-sample results for 320 comparisons by crossing four levels of fine tuning, four levels of image noise, and 20 CNN pairs.

The shaded area in Fig. 4 shows that there is a relatively narrow band of performance difference that produces complementarity (see *SI Appendix* for computational details). The human and machine classifiers need to perform at similar levels in order to produce a hybrid HM pair that is more accurate than either two humans or two machine classifiers. These results strongly depend on the correlations between human and machine classifier. For example, in a hypothetical scenario where the HM classifier correlation is zero, the zone of complementarity will grow (dashed line). However, note that even in this best-case scenario, there are still limits on the accuracy differences that produce complementarity.

### Differentiating between Human and Machine Classifier Errors and Confidence Scores Improves Hybrid HM Performance.

In our fourth and final finding, we consider how the performance of the hybrid HM pairs depends on a number of combinations of different factors: 1) the presence of a class-specific error model that can correct for human and machine-classifier errors and biases for individual labels, 2) the presence of human confidence scores, and 3) the presence of machine classifier scores. Table 1 shows the out-of-sample accuracy of a hybrid pair when systematically varying these three factors. See *SI Appendix* for details on models and experimental methodology. The results are averaged over the five machine classifiers (*SI Appendix* shows results broken down by individual classifiers). Each of the three factors contributes to an improvement in performance of the hybrid ensemble, especially for high-noise conditions. In addition, each of these factors has an independent effect on hybrid performance. Table 2 shows the statistical analysis of the relative effects of the three factors on hybrid performance. All three factors are significant. The availability of machine confidence has a larger effect on performance than either the availability of human confidence or an error model. The difference in confidence scoring likely contributes to this difference—the machine classifiers express confidence scores across all labels simultaneously whereas the human participants express only a single confidence score associated with the decision. In addition, the human confidence and error model contribute about the same in performance to hybrid performance. Thus, a simple way to boost performance of hybrid HM classifiers is to elicit human confidence ratings.

**Table 1.** Accuracy for HM classifier combinations across image noise and different types of combination models that vary the presence or absence of an error model, human confidence scores, and machine classifier confidence scores

** **			Image noise (*ω*)			
Error	Human	Machine				
model	confidence	confidence	80	95	110	125
*✓*	*✓*	*✓*	0.933	0.906	0.850	0.748
X	*✓*	*✓*	0.927	0.899	0.841	0.722
*✓*	X	*✓*	0.928	0.902	0.844	0.738
X	X	*✓*	0.925	0.895	0.830	0.707
*✓*	*✓*	X	0.911	0.883	0.823	0.701
X	*✓*	X	0.903	0.876	0.815	0.686
*✓*	X	X	0.901	0.872	0.805	0.674
X	X	X	0.895	0.858	0.769	0.636

The results are averaged across the five machine classifiers. Each accuracy result is based on 36,000 observations.

**Table 2.** Effect of including class-specific error model, human confidence, and machine classifier confidence scores on hybrid HM performance

	Accuracy (log odds)		
Predictors	Estimate	CI	*P*
Intercept	1.368	1.358–1.377	<0.001
Error model	0.101	0.091–0.110	<0.001
Human confidence	0.104	0.094–0.114	<0.001
Machine confidence	0.257	0.247–0.266	<0.001
Observations	1,152,000		

## Discussion

Previous work has shown the benefits of separately combining the predictions of diverse machine classifiers (39–42) or groups of people (34–38). In this work, we extend these results by systematically investigating the factors that influence the performance of hybrid combinations of machine and human classifiers. We collected a large-scale behavioral and machine classifier dataset where both humans and machine classifiers make predictions for the same data. The results showed that even if performance from a human exceeds the performance of a machine classifier, adding the predictions from the machine classifier to a single human can lead to better performance than combining the predictions of two humans. The converse is also true. Even if a machine classifier outperforms humans, a hybrid HM pair can still outperform the predictions from a combination of machine classifiers that are all individually outperforming a single human.

Our results have implications for algorithmic systems that have not yet achieved human-level accuracy (53). Starting with a human predictor, adding algorithmic predictions (that are less accurate than the human) may be more beneficial than adding additional human predictors. Thus, the benchmark for evaluating AI algorithms need not necessarily be human-level performance. If an algorithm does not achieve human-level accuracy, it can still lead to increased accuracy in combined hybrid predictions. Conversely, our results also indicate that once AI approaches have exceeded human performance in particular domains, this does not imply that human judgment is no longer useful in hybrid HM systems.

However, there are limits to the scope of complementarity. Prior work has shown empirically that hybrid HM algorithm systems do not always lead to superior performance (33, 54, 55). Our results in this paper go beyond these earlier studies, both theoretically and empirically, and show specifically what factors contribute to complementarity (25). In particular, the key limiting factor for complementarity is the degree of correlation between human and machine classifier predictions. A large correlation leads to limits on the accuracy difference between classifiers that can support complementarity. This result has implications for human–AI collaborative settings where algorithms are used as decision aids (55, 56). Effective AI advice should not only be accurate but also be as independent as possible from human judgment. Independence of the AI component from the human could, for example, be increased by leveraging different mechanisms to produce predictions or changing the objective function for the AI model (31). Interestingly, the goal of decreasing the correlation between human and algorithmic predictions stands in contrast with modeling natural intelligence, where the goal is to create computational models that mimic human internal processing mechanisms (28).

Another important factor is the role of both human and machine classifier confidence scores. While machine classifier scores have been used before in hybrid HM systems (57), human confidence is often not elicited (29, 58). However, our results show that human confidence ratings can significantly increase hybrid performance and are as effective in improving combined performance as inferring an explicit error model that can correct for class-specific errors and biases. Confidence scores allow differing abilities of human and machine classifiers to be resolved at the level of individual instances.

Overall, our results add to a growing literature showing the advantages of combining human and AI predictions in areas such as crowdsourcing (29, 58, 59), providing a framework for assessing hybrid combinations of human and machine predictions, with potential applications in high-stakes domains such as medicine (60–62) and the justice system (63, 64).

## Materials and Methods

### Images for Experiments.

There are 1,200 unique images total in our dataset, divided equally into 16 classes (chair, oven, knife, bottle, keyboard, clock, boat, bicycle, airplane, truck, car, elephant, bear, dog, cat, and bird). The images and categories are based on a subset of the ImageNet Large Scale Visual Recognition Challenge (ILSRVR) 2012 database (65). As ground truth labels we used the original labels from the ILSRVR database. To create a more challenging classification task for both the human participants and machine classifiers, images were distorted by phase noise at each spatial frequency, where the phase noise is uniformly distributed in the interval [−ω,ω] (66). Four levels of phase noise, ω={80,95,110,125}, were applied to each of the 1,200 unique images, resulting in 4,800 images (see *SI Appendix*, Fig. S3 for examples).

### Behavioral Image Classification Experiment.

The behavioral image classification dataset consists of 28,997 human classifications from a total of 145 participants. The experimental protocol was approved by University of California, Irvine Institutional Review Board. Informed consent was obtained from participants before continuing to the classification task. Each participant classified 200 images into the 16 categories. For each classification, participants also provided a discrete confidence level (low, medium, or high). The behavioral classification dataset contains at least six human classifications for each of the 4,800 images. Human performance decreases as a function of image noise (*SI Appendix*, Fig. S4), and accuracy varies systematically as a function of expressed confidence, showing that confidence is related to decisional uncertainty.

### Machine Classifier Predictions.

We created a set of machine classifier predictions for the 4,800 images and the set of 16 classes in the behavioral dataset. For each image, each classifier produces a probability vector over the 16 classes, containing the confidence scores for each class. The class associated with the highest probability corresponds to the classification for the image. To vary performance of the machine classifiers relative to human performance, we selected five different machine classifiers pretrained for ImageNet: AlexNet (67), DenseNet161 (68), GoogleNet (69), ResNet152 (70), and VGG-19 (71). To create additional levels of performance variation, we retrained the models to varying degrees to adapt to the image distortions. For each of the five classifiers, we retrained four variants of each model, based on how many passes through the noisy images data (epochs) are used during stochastic gradient training, producing in effect four variants that are adapted/fine-tuned to varying degrees of noise. The models were fine-tuned for either 0 epochs (baseline), between 0 and 1 epochs, 1 epoch, and 10 epochs. The second level of fine tuning (0 to 1 epochs) was based on a checkpoint during training before 1 epoch was reached, leading to a performance level intermediate between baseline and 1 epoch of training. The different machine classifiers produce a variety of performance levels relative to human performance, with some fine-tuned VGG-19 and DenseNet161 classifiers exceeding human performance at the high image distortion levels (*SI Appendix*, Fig. S4).

## Supplementary Material

Supplementary File

## Data Availability

The analysis code and data are available at Open Science Foundation (OSF) (https://osf.io/2ntrf/?view_only=9ec9cacb806d4a1ea4e2f8acaada8f6c).
