# Supplementary material for: Bayesian modeling of human–AI complementarity
Source: Proc Natl Acad Sci U S A. 2022 Mar 11;119(11):e2111547119. doi: 10.1073/pnas.2111547119 (PMC8931210; doi:10.1073/pnas.2111547119)
Supplement: Supplementary File [file pnas.2111547119.sapp.pdf]

PNAS
www.pnas.org

Supplementary Information for

Bayesian Modeling of Human-AI Complementarity

Mark Steyvers, Heliodoro Tejeda, Gavin Kerrigan and Padhraic Smyth

Mark Steyvers.
E-mail: mark.steyvers@uci.edu

This PDF file includes:

Supplementary text
Figs. S1 to S11 (not allowed for Brief Reports)
Tables S1 to S2 (not allowed for Brief Reports)
SI References

Supporting Information Text

Methods

Images for Experiments. All images used in this study come from the ImageNet Large Scale Visual Recognition Challenge (ILSRVR) 2012 database (1). The training set from this database contains a total of 1,281,167 images corresponding to 1000 image categories. For the purpose of the human classification experiment, we created a subset of 16 categories (chair, oven, knife, bottle, keyboard, clock, boat, bicycle, airplane, truck, car, elephant, bear, dog, cat, and bird). This 16-class-ImageNet was created following a similar procedure as in (2) by mapping the 16 categories to subsets of the 1000 ImageNet categories using a hierarchical mapping from WordNet (e.g. the bear category combined ImageNet classes such as brown bear, American black bear, ice bear, and sloth bear). In this mapping, 207 of the 1000 ImageNet classes were used for the 16 class dataset. From the subset of 262,369 16-class-ImageNet images, we randomly selected 75 images per category, resulting in 1200 unique images for the human classification experiments.

To vary the degree of difficulty in the human and machine classifier experiments, we applied phase noise distortion to the images (2, 3). The phase noise distortion was applied at each spatial frequency, uniformly distributed in the interval $[-\omega, \omega]$. Four levels of phase noise, $\omega = \{80, 95, 110, 125\}$, were applied to each of the 1200 unique images resulting in 4800 images. Given that images in the ILSRVR 2012 database are of different sizes and shapes, we preprocessed the images by first selecting the largest central square region of the image, resizing the image to 224 x 224 pixels, and finally applying the phase noise transform. Examples of the resulting preprocessed images at the four image distortion levels are shown in Figure S3.

Human Classification Data. We collected human classifications data from 145 participants from Amazon Mechanical Turk. After obtained informed consent, participants were instructed to classify noisy images as accurately as possible into 16 categories. At the start of each trial, a noisy image was shown, and the participant selected their response by clicking one of 16 response icons, arranged in a 4 x 4 grid. In contrast to (2), the image was presented for the entire duration of the trial and response confidence was assessed. After selecting the category, participants indicated their response confidence by selecting one of three confidence levels, “low”, “medium”, and “high”. No feedback was provided after selecting a category and submitting a confidence response. Each participant classified a total of 200 images. For each participant, the images were randomly selected from the set of 4800 images with the constraint that each unique image could only be shown once and that the four noise levels were equally represented across the 200 trials. The resulting human classification dataset consists of 28,997 classifications containing (at least) six human classifications for each of the 4800 images.

Before being eligible to participate in the study, all participants were given instructions of the task and were required to pass a comprehension check by classifying four out of five images correctly. They were given two attempts to accomplish this. Only participants who successfully completed the comprehension check were allowed to continue with the study. Participants took a median of 24 minutes for the classification phase of the experiment. A payment of $6 USD was provided through Amazon Mechanical Turk, for successful completion of the study.

Machine Classifier Predictions. We created a set of machine classifiers with varying degrees of classification performance (relative to human performance) by selecting different types of machine classifiers and applying to each classifier a variable degree of fine-tuning to the image noise. The set of classifiers included 5 pre-trained ImageNet models: AlexNet (4), DenseNet161 (5), GoogleNet (6), ResNet152 (7), and VGG-19 (8). All models are based on implementations provided by PyTorch. Before any fine-tuning, the pretrained classifiers showed poor performance for the noisy images even at the lowest noise level. To fine-tune, the pretrained model was loaded and default ImageNet training parameters for that model were used. Given that the models were initially trained using all 1000 ImageNet categories, we decided to fine-tune each model using the entire ILSRVR 2012 database, excluding the 1200 images selected for this study. During the fine-tuning process, each particular batch of images was distorted by random phase noise that ranged from 0 to 130 in increments of 5. For each type of classifier, we varied the amount of fine-tuning to the image noise across four levels. The models were finetuned for either 0 epochs (baseline), between 0 and 1 epochs, 1 epoch, and 10 epochs. The second level of finetuning (0-1 epochs) is based on a checkpoint during training before 1 epoch was reached that led a performance level intermediate between baseline and 1 epoch of training.

The output of the classifiers after fine-tuning is based on the softmax probabilities for 1000 ImageNet classes. This output was transformed to a probability vector for the 16 classes by taking the maximum ImageNet class probability corresponding to each of the 16 classes, and renormalizing. For example, to create a score for the bear category in our 16 classes set, we took the maximum score from the set of four ImageNet classes that were mapped to the bear category.

Training and Validation data for Classifier Pairs. For the Bayesian combination model, we created a number of data sets based on three different types of pairs: human-human, human-machine, and machine-machine classifier pairs. The datasets were constructed with the following constraints: 1) only a single level of image distortion was used within each dataset, 2) each unique image occurs only a single time within each dataset, 3) only one type of machine classifier at a single level of fine-tuning is used within each dataset. Based on these constraints, we created 12 datasets for the homogeneous human-human pairs by combining four image noise levels with three ways to create random pairs for each image (note that each image had classifications from at least six different human participants). For the heterogeneous human-machine classifier pairs, we created 360 datasets by combining 5 types of machine classifiers, 3 levels of fine-tuning, 4 image noise levels, with 6 different human participants (the 6 participants corresponded to either the first or second of the human participant pairs in the human-human

data sets). Finally, for the homogeneous machine-machine classifier pairs, we created 120 data sets by combining 10 pairing of machine classifiers, 3 levels of fine-tuning, with 4 levels of image noise.

Each dataset constructed involved exactly 1200 predictions (corresponding to 1200 unique images). The datasets were split into four random partitions for the purpose of four-fold cross-validation. Therefore, in any particular cross-validation partition, 800 predictions were used to train the Bayesian combination model (i.e., the true class labels were observed) and 400 predictions were used for validation.

Model Details

Ordered Probit Model. To model the human confidence ratings, we use an ordered probit model that probabilistically maps the latent probability score γ_{i,y_i} corresponding to the classification made by the human to an ordinal confidence rating, r_i. For our data, we have three confidence ratings (1="Low", 2="Medium", and 3="High") generated according to:

$$r_i \sim \text{OrderedProbit}(\gamma_{i,y_i}, c, \delta) \qquad [1]$$

The ordered probit model is constructed in the following way:

$$\begin{aligned} x_{i,1} &= \Phi(\delta(c_1 - \gamma_{i,y_i})) \\ x_{i,2} &= \Phi(\delta(c_2 - \gamma_{i,y_i})) - \Phi(\delta(c_1 - \gamma_{i,y_i})) \\ x_{i,3} &= 1 - \Phi(\delta(c_2 - \gamma_{i,y_i})) \\ r_i &\sim \text{Categorical}(x_{i,1}, x_{i,2}, x_{i,3}) \end{aligned} \qquad [2]$$

where Φ is the cumulative standard normal distribution, and x_1, x_2, and x_3 represent the latent probabilities for producing a low, medium, or high confidence rating. The two cutpoint parameters c_1 and c_2 determine the intervals that map the latent confidence score into a confidence rating. The δ parameter determines the sharpness of the rating probability curves (i.e., the degree of randomness in the probabilistic mapping from the confidence score to a rating). Figure S2 shows an example of how the latent probabilities are mapped to three ordinal ratings ("high", "medium", and "low") for two values of δ and criterion values $c_1 = 0.2$ and $c_2 = 0.4$. Note that δ controls the degree of noise in mapping from latent probabilities to ordinal ratings.

Computing the zone of complementarity. For the predictions in Fig. 4, we assume $\rho_{HM} = 0.33$, $\rho_{HH} = 0.62$, and $\rho_{MM} = 0.71$, approximately matching the correlations inferred by the Bayesian combination model. We use numerical methods to find the zone of complementarity represented by the red area in Fig. 4. The variable a_H is varied between 0.1 and 5 in 40 steps. For each value of a_H, the lower bound of a_M that produces complementarity is identified by finding the roots of the function $r_{HM} - r_{HH}$ using Eq. 6. Next, we find the upper bound of a_M that produces complementarity by finding the roots of the function $r_{MM} - r_{HM}$. We apply Eq. 5 to the a_H and a_M pairs to find the coordinates A_H and A_M in Fig. 4.

Assessing the effect of a class-specific error model and presence of confidence scores. In Table 1 (full results shown in Table S2), we consider how the performance of the hybrid human-machine pairs depends on a number of combinations of different factors. First, we consider the presence of a *class-specific error-model* that can correct for human and machine-classifier specific errors and biases for individual labels. For example, relative to a machine classifier, a human might be better at discriminating a particular label from other labels or might display a response bias for some labels such that that those labels are predicted more often than expected by chance. In the error model extension, means a and b in Eq. 1 become class-specific but not the covariance. The second factor is the presence of *human confidence* scores. If human confidence ratings are present, we apply the model with the generative process for human confidence ratings as specified by Eq. 4 Otherwise, Eq. 4 is left out of the model. Finally, the third factor is the presence of *machine confidence* scores. If the machine classifier confidence scores are present, the logit scores for the machine classifier in Eq. 1 are observable. Otherwise, the logit scores become latent variables and Eq 3 is used to model the classification from the machine classifier.

For each particular combination of these three factors, we applied the model separately to each of the 5 CNNs and 4 image noise levels. Each entry in Table 1 is based on 36,000 observations by combining the results across CNNs (5), unique images (1200) and unique human participants per image (6).

Model Inference. For posterior inference, we used a JAGS Markov chain Monte Carlo sampler (9) and ran the sampler with 8 chains with a burnin of 1000 iterations before taking 50 samples per chain. The chains mixed appropriately. For each instance, the mode of the latent classifications z across the 400 samples was used to determine the aggregate classifications $\hat{z}$.

For prior distributions, we place a uniform prior on the latent true label, $z_i \sim \text{Uniform}(\{1, ..., L\})$. This prior can be replaced by other priors to allow for skew in the label distribution. For the correlation between classifiers, we used $\rho \sim \text{Uniform}(-1, 1)$. For the machine classifiers, we use priors: $a \sim \mathcal{N}(0, 10), b \sim \mathcal{N}(0, 10), \sigma \sim \text{Uniform}(0, 15)$. For human classifications, the same priors were used but with added constraints $b = 0$ and $\sigma = 1$ for the purpose of identifiability. In addition, we used $\delta \sim \text{Uniform}(0, 100)$ for the scaling parameter and uniform priors on the cutpoints, $c \sim \text{Uniform}(0, 1)$, with the constraint that the cutpoints are ordered (i.e. $c_r < c_{r+1}$ for $r = 1, .., R - 1$). Finally, after experimenting with a number of values for τ, we set $\tau = 0.05$ for best convergence results.

Additional Results

Distribution of Machine Classifier Logit Scores. Supplemental Figure S5 shows the empirical distributions of the λ logit scores for correct and incorrect classes for the 16-class ImageNet dataset. The distributions are approximately normal (with some left and right skew for the incorrect and correct label distributions).

Pattern of class-specific errors by human and machine classifiers. Some of the differences between human and machine classifiers can be summarized by looking at the pattern of correct and incorrect classifications at the level of individual classes. Figure S8 shows the class-wise confusion matrices for humans and each of the four machine classifier for the most challenging level of image distortion in the experiment. The machine classifiers are fine-tuned for one epoch. The machine classifier VGG-19, for example, makes more correct classifications for classes such as truck, dog and bird, whereas the human makes more correct classifications for the car class. In addition, there are a number of class-confusions that are more prevalent in the machine classifier relative to humans (e.g., confusing cats with dogs). These results show that human and machine classifiers make different types of errors at the class level.

To further evaluate the class-specific errors, we analyze the parameters inferred by the class-specific error model. Specifically, we assess a discrimination score $d_j = (a_j - b_j)/\sigma$ for each label j. This score represents the separation between the logit scores for the correct and incorrect label normalized by the standard deviation. This score determines the ability of the classifier to discriminate between that label and all other labels, analogous to the discriminability index in signal detection theory (10). The baseline parameter b_j determines the response bias for label j. If b_j is relatively high for one particular label, the model predicts higher confidence scores and a larger number of responses (a priori) for that label. To facilitate interpretation, we will report mean centered b values (i.e., $\sum_j b_j = 0$).

Table S1 shows the resulting estimates of discrimination and bias scores when the model extension is applied to a hybrid ensemble of a single human and the VGG-19 classifier. Across image noise levels, the VGG-19 classifier is biased towards the labels "dog", "truck", and biased away from "airplace" and "knife" whereas the human participants reveal small response biases toward "boat", "car", and "dog" and away from "knife". In terms of the relative discrimination ability (i.e., $d_H - d_M$), the human participants are better able to detect the "car", "clock", and "knife" labels relative to VGG-19, whereas the CNN classifier is relatively good at detecting "boat" and "bird". Overall, the results show systematic differences between human and machine classifiers in terms of response biases and ability to discriminate between individual classes.

Robustness to confidence scoring. One potential contributing factor to complementarity is the difference in the type and amount of information available from machine and human classifier. The machine classifier provides a full set of confidence scores across all classes whereas the human classifier provides only a single confidence score (associated with the classification made for the instance). In addition, the machine classifier scores are continuous whereas the human confidence score is discrete (three responses, "high", "medium", and "low").

To verify that our findings are robust to changes in the way confidence scores are produced, we also applied the Bayesian combination model when the machine classifier confidence score are only observed for the winning class for each instance and the scores are discretized to three bins (analogous to the three confidence levels for the human classifiers). The discretization was performed to create uniform distributions of responses across the three bins. With this procedure, human and machine classifier provide the same type of confidence scores.

Supplemental Figures S6 and S7 show the results. To facilitate comparison, the original results (with fully observed and continuous machine classifier scores) are shown in the bottom half of the Figures along with the new results in the top half. The results show that the hybrid pair performance with partially observed, discretized machine classifier scores are somewhat lower relative to pairs with the full machine classifier information and fewer hybrid classifiers exceed the performance of two humans. However, the overall pattern of accuracy is qualitatively similar. Critically, the pattern of correlations is qualitatively the same. Hybrid combinations of classifiers produce the lowest correlations, and machine-only combinations produce the highest correlations. As expected, posterior uncertainty for pairs of machine classifier combinations has increased due to the decrease in available confidence scores.

Therefore, these results show that human-machine classifier complementarity is in part influenced by the type of confidence scores available. Having a full set of continuous confidence scores contributes to improved pair performance.

Theoretical Analysis

Predicting complementarity. Given the structure and parameters of our Bayesian model, we derive expressions for the accuracy of individual classifiers as well as combinations of two classifiers. This allows us to analytically determine the conditions that lead to complementarity.

Consider a set of two human and two model classifiers $\mathcal{C} = \{H_1, H_2, M_1, M_2\}$. Let $C_1, C_2 \in \mathcal{C}$ be any two classifiers selected from this set. The accuracy of a single classifier $C \in \mathcal{C}$ is denoted A_C, and the accuracy of the Bayesian combination model derived from the pair of classifiers C_1 and C_2 is denoted A_{C_1,C_2}. Recall that we have complementarity if $A_{HM} > \max\{A_{H_1H_2}, A_{M_1M_2}\}$ for some $H \in \{H_1, H_2\}$ and some $M \in \{M_1, M_2\}$.

We make the following assumptions on the parameters of our Bayesian models to simplify our analysis: (i) $b_C = 0$ for each $C \in \mathcal{C}$; (ii) The Bayesian model parameters for the humans are equal, i.e. $a_{H_1} = a_{H_2} =: a_H$ and $\sigma_{H_1} = \sigma_{H_2} =: \sigma_H$; (iii) The Bayesian model parameters for the machine classifiers are equal, i.e. $a_{M_1} = a_{M_2} =: a_M$ and $\sigma_{M_1} = \sigma_{M_2} =: \sigma_M$;

(iv) The marginal distribution over classes is uniform, i.e. the marginal probability of seeing class i is $p(z = i) = 1/L$ for $i \in \{1, 2, \ldots, L\}$.

Assumption (ii) implies that H_1 and H_2 are exchangeable under our Bayesian model (respectively for assumption (iii) and M_1, M_2). Without loss of generality, we can additionally assume that the true class is the first one. We use ρ_{HH}, ρ_{MM} to denote the correlation parameter between the human (respectively model) labelers above and ρ_{HM} to denote the correlation between any human with any machine classifier.

An illustrative special case: $L = 2$ ***classes.*** To demonstrate our analysis, we begin with the special case of binary ($L = 2$) classification.

For an individual classifier C, there are two logit scores sampled in the model, $\lambda_1 \sim \mathcal{N}(a, \sigma)$ and $\lambda_2 \sim \mathcal{N}(0, \sigma)$, associated with the correct and incorrect class respectively. The accuracy for this classifier conditional on model parameters is

$$\begin{aligned} A_C &= p\{z = y | a, \sigma\} \\ &= p\{\phi(\lambda_1 | a, \sigma)\phi(\lambda_2 | 0, \sigma) > \phi(\lambda_1 | 0, \sigma)\phi(\lambda_2 | a, \sigma)\} \\ &= p\{\lambda_1 > \lambda_2\} \\ &= p\{\lambda_1 - \lambda_2 > 0\} \\ &= \Phi\left(\frac{1}{\sqrt{2}}\frac{a}{\sigma}\right) \end{aligned} \tag{3}$$

where $\phi(\lambda|\mu, \sigma)$ is the normal density for x given mean μ and standard deviation σ and Φ denotes the cumulative distribution function for the standard normal distribution. Hence, for two individual classifiers, C_1 and C_2, we will have $A_{C_1} > A_{C_2}$ if and only if $\frac{a_1}{\sigma_1} > \frac{a_2}{\sigma_2}$.

For two classifiers, we have two pairs of logit scores. For the correct and incorrect class, the pairs of logit scores are sampled from the bivariate normals, $\binom{\lambda_{1,1}}{\lambda_{1,2}} \sim \mathcal{N}\left[\binom{a_1}{a_2}, \Sigma\right]$ and $\binom{\lambda_{2,1}}{\lambda_{2,2}} \sim \mathcal{N}\left[\binom{0}{0}, \Sigma\right]$ respectively, with covariance matrix $\Sigma = \begin{bmatrix} \sigma_1^2 & \sigma_1\sigma_2\rho \\ \sigma_1\sigma_2\rho & \sigma_2^2 \end{bmatrix}$. The accuracy for the combined classifier is then

$$\begin{aligned} A_{C_1,C_2} &= p\{z = y | a_1, a_2, \sigma_1, \sigma_2, \rho\} \\ &= p\left\{\phi\left(\tbinom{\lambda_{1,1}}{\lambda_{1,2}} | \tbinom{a_1}{a_2}, \Sigma\right)\phi\left(\tbinom{\lambda_{2,1}}{\lambda_{2,2}} | \tbinom{0}{0}, \Sigma\right) > \phi\left(\tbinom{\lambda_{1,1}}{\lambda_{1,2}} | \tbinom{0}{0}, \Sigma\right)\phi\left(\tbinom{\lambda_{2,1}}{\lambda_{2,2}} | \tbinom{a_1}{a_2}, \Sigma\right)\right\} \\ &= p\{\lambda_{1,1}(a_2\rho - a_1) + \lambda_{1,2}(a_1\rho - a_2) > \lambda_{2,1}(a_2\rho - a_1) + \lambda_{2,2}(a_1\rho - a_2)\} \\ &= \Phi\left(\frac{a_1^2 + a_2^2 - 2a_1a_2\rho}{\sqrt{2\sigma_1^2(a_2\rho - a_1)^2 + 2\sigma_2^2(a_1\rho - a_2)^2 + 4\rho\sigma_1\sigma_2(a_2\rho - a_1)(a_1\rho - a_2)}}\right) \end{aligned} \tag{4}$$

In order to facilitate the study of complementarity, we specialize the above results to the case of homogeneous and heterogeneous pairs.

For the sake of simplicity, we describe the homogeneous analysis in the case of an pair of two humans H_1 and H_2. The analysis can translated to that of homogeneous pairs of models by making the necessary changes in notation.

In this case, under the set of assumptions outlined above, by simplifying Equation Eq. (4) we can express the pair accuracy as

$$A_{H_1,H_2} = \Phi\left(\frac{1}{\sqrt{1 + \rho_{HH}}}\frac{a_H}{\sigma_H}\right) \tag{5}$$

where $\rho_{HH} \in [0, 1]$ is the human-human correlation. As we would expect, as ρ_{HH} increases, the accuracy of the pair will decrease.

To illustrate these results, we compare to the accuracy of a single human. By Equation Eq. (3) and Eq. (4), we have $A_{H_1,H_2} > A_{H_i}$ when

$$\frac{a_H}{\sigma_H}\frac{1}{\sqrt{1 + \rho_{HH}}} > \frac{1}{\sqrt{2}}\frac{a_H}{\sigma_H} \tag{6}$$

Note that $\rho_{HH} \leq 1$, so that this inequality will always hold, i.e. under our assumptions the pair of two humans will always have a higher accuracy than a single human.

In the case of a heterogeneous pair consisting of a human labeler and model labeler, assume further that the models and humans have the same variance, i.e. $\sigma := \sigma_H = \sigma_M$. We can express the heterogeneous human-model pair accuracy A_{HM} as

$$A_{HM} = \Phi\left(\frac{1}{\sqrt{2}\sigma}\frac{1}{\sqrt{1 + \rho_{HM}}}\sqrt{\frac{a_H^2 + a_M^2 - 2a_Ha_M\rho_{HM}}{1 - \rho_{HM}}}\right) \tag{7}$$

Conditions for complementarity. We derive a necessary and sufficient condition on the correlation ρ_{HM} to achieve complementarity. Since $\Phi(\cdot)$ is a strictly increasing function, it suffices to compare the arguments of Equation Eq. (5) and Equation Eq. (7). Doing so, we have $A_{HM} > A_{HH}$ if and only if

$$\frac{1}{1+\rho_{HM}}\left(\frac{1}{2}\frac{a_H^2+a_M^2-2a_Ha_M\rho_{HM}}{1-\rho_{HM}}\right) > \frac{1}{1+\rho_{HH}}a_H^2$$

and we similarly have $A_{HM} > A_{MM}$ if and only if

$$\frac{1}{1+\rho_{HM}}\left(\frac{1}{2}\frac{a_H^2+a_M^2-2a_Ha_M\rho_{HM}}{1-\rho_{HM}}\right) > \frac{1}{1+\rho_{MM}}a_M^2$$

Hence, we have complementarity if and only if both of the previous inequalities are satisfied, which in turn is equivalent to

$$\frac{a_H^2+a_M^2}{2a_Ha_M} = \frac{1}{2}\left(\frac{a_H}{a_M}+\frac{a_M}{a_H}\right) > \rho_{HM} + \left(1-\rho_{HM}^2\right)\max\left\{\frac{a_H}{a_M}\frac{1}{1+\rho_{HH}}, \frac{a_M}{a_H}\frac{1}{1+\rho_{MM}}\right\} \tag{8}$$

This is quadratic in ρ_{HM}, allowing us to solve for the conditions on ρ_{HM} that will lead to complementarity.

Complementarity for $L \geq 2$ classes. In this section, we derive expressions for the accuracy of an individual classifier and for the accuracy of our Bayesian pair in the more general multi-class classification setting.

For an individual classifier C, one of the logit scores, $\lambda_1 \sim \mathcal{N}(a,\sigma)$ corresponds to the correct class. For the remaining $j = \{2, ..., L\}$ classes, the logit score for the incorrect class is $\lambda_j \sim \mathcal{N}(0,\sigma)$. To make a correct prediction in this setup would mean that $\lambda_1 > \lambda_j$ for $j = 2, 3, \ldots, L$, i.e. the score for the correct label is greater than the score for every other class.

$$\begin{aligned}
A_c &= p\{z = y|a,\sigma\} \\
&= p\{\phi(\lambda_1|a,\sigma)\phi(\lambda_k|0,\sigma)\prod_{j\neq 1,k}\phi(\lambda_j|0,\sigma) > \phi(\lambda_1|0,\sigma)\phi(\lambda_k|a,\sigma)\prod_{j\neq 1,k}\phi(\lambda_j|0,\sigma) \qquad \forall k = 2,\ldots,L\} \\
&= p\{\phi(\lambda_1|a,\sigma)\phi(\lambda_k|0,\sigma) > \phi(\lambda_1|0,\sigma)\phi(\lambda_k|a,\sigma) \qquad \forall k = 2,\ldots,L\} \\
&= p\{\lambda_1 > \lambda_k \qquad \forall k = 2,\ldots,L\} \qquad \text{(by Equation Eq. (3))} \\
&= \int_{-\infty}^{\infty}\Phi\left(x\right)^{L-1}\phi\left(x-\frac{a}{\sigma}\right)dx
\end{aligned} \tag{9}$$

We can perform a similar analysis for the pair of C_1 and C_2:

$$\begin{aligned}
A_{C_1,C_2} &= p\{z = y|a_1,a_2,\sigma_1,\sigma_2,\rho\} \\
&= p\{\phi\left(\left(\begin{smallmatrix}\lambda_{1,1}\\ \lambda_{1,2}\end{smallmatrix}\right)|\left(\begin{smallmatrix}a_1\\ a_2\end{smallmatrix}\right),\Sigma\right)\prod_{j=2}^{L}\phi\left(\left(\begin{smallmatrix}\lambda_{j,1}\\ \lambda_{j,2}\end{smallmatrix}\right)|\left(\begin{smallmatrix}0\\ 0\end{smallmatrix}\right),\Sigma\right) > \\
&\qquad \phi\left(\left(\begin{smallmatrix}\lambda_{1,1}\\ \lambda_{1,2}\end{smallmatrix}\right)|\left(\begin{smallmatrix}0\\ 0\end{smallmatrix}\right),\Sigma\right)\phi\left(\left(\begin{smallmatrix}\lambda_{k,1}\\ \lambda_{k,2}\end{smallmatrix}\right)|\left(\begin{smallmatrix}a_1\\ a_2\end{smallmatrix}\right),\Sigma\right)\prod_{j\neq 1,k}^{L}\phi\left(\left(\begin{smallmatrix}\lambda_{j,1}\\ \lambda_{j,2}\end{smallmatrix}\right)|\left(\begin{smallmatrix}0\\ 0\end{smallmatrix}\right),\Sigma\right) \qquad \forall k = 2,\ldots,L\} \\
&= p\{\phi\left(\left(\begin{smallmatrix}\lambda_{1,1}\\ \lambda_{1,2}\end{smallmatrix}\right)|\left(\begin{smallmatrix}a_1\\ a_2\end{smallmatrix}\right),\Sigma\right)\phi\left(\left(\begin{smallmatrix}\lambda_{k,1}\\ \lambda_{k,2}\end{smallmatrix}\right)|\left(\begin{smallmatrix}0\\ 0\end{smallmatrix}\right),\Sigma\right) > \phi\left(\left(\begin{smallmatrix}\lambda_{1,1}\\ \lambda_{1,2}\end{smallmatrix}\right)|\left(\begin{smallmatrix}0\\ 0\end{smallmatrix}\right),\Sigma\right)\phi\left(\left(\begin{smallmatrix}\lambda_{k,1}\\ \lambda_{k,2}\end{smallmatrix}\right)|\left(\begin{smallmatrix}a_1\\ a_2\end{smallmatrix}\right),\Sigma\right) \qquad \forall k = 2,\ldots,L\} \\
&= p\{\lambda_{1,1}(a_2\rho - a_1) + \lambda_{1,2}(a_1\rho - a_2) > \lambda_{k,1}(a_2\rho - a_1) + \lambda_{k,2}(a_1\rho - a_2) \qquad \forall k = 2,\ldots,L\} \\
&= \int_{-\infty}^{\infty}\Phi\left(x\right)^{L-1}\phi\left(x - \frac{a_1^2+a_2^2-2a_1a_2\rho}{\sqrt{\sigma_1^2(a_2\rho-a_1)^2+\sigma_2^2(a_1\rho-a_2)^2+2\rho\sigma_1\sigma_2(a_2\rho-a_1)(a_1\rho-a_2)}}\right)dx
\end{aligned} \tag{10}$$

We can use the above integral forms to derive an if and only if condition for complementarity. Let r_{C_1,C_2} be the ratio that appears in the argument of $\phi(\cdot)$ in Equation Eq. (10):

$$r_{C_1,C_2} = \frac{a_1^2+a_2^2-2a_1a_2\rho}{\sqrt{\sigma_1^2(a_2\rho-a_1)^2+\sigma_2^2(a_1\rho-a_2)^2+2\rho\sigma_1\sigma_2(a_2\rho-a_1)(a_1\rho-a_2)}} \tag{11}$$

Under our assumptions, this ratio can be simplified to a more interpretable form in the hybrid and non-hybrid cases:

$$\begin{aligned}
r_{H_1,H_2} &= \frac{|a_H|}{\sigma_H}\sqrt{\frac{2}{1+\rho_{HH}}} \qquad r_{M_1,M_2} = \frac{|a_M|}{\sigma_M}\sqrt{\frac{2}{1+\rho_{MM}}} \\
r_{HM} &= \frac{1}{\sigma\sqrt{1-\rho_{HM}}}\sqrt{\frac{a_H^2+a_M^2-2a_Ha_M\rho_{HM}}{1+\rho_{HM}}}
\end{aligned} \tag{12}$$

The following claim shows that complementarity can be determined entirely by the r terms above. Note that this ratio is the same as the argument of $\Phi(\cdot)$ in Equation Eq. (4), up to a constant factor of $\sqrt{2}$. As we studied this extensively in the binary case, we then see that complementarity in the multi-class case reduces to complementarity in the binary case.

Claim: We have complementarity if and only if $r_{HM} > \max\{r_{HH}, r_{MM}\}$.

Proof. We prove that $r_{HM} > r_{HH}$ is sufficient for $A_{HM} > A_{HH}$. The proof for the pair of two models is analogous, and so $r_{HM} > \max\{r_{HH}, r_{MM}\}$ will satisfy $A_{HM} > A_{HH}$ and $A_{HM} > A_{MM}$ simultaneously. The same argument also works (with minor modifications) to prove the "only if" part of the statement.

Set $\Delta_H = r_{HM} - r_{HH}$. We have $\Delta_H > 0$ by assumption. We can evaluate the accuracies with the above formulae and use a change of variables to prove the claim:

$$
\begin{aligned}
A_{HM} &= \int_{-\infty}^{\infty} \Phi(x)^{L-1}\phi(x - r_{HM})\, dx \\
&= \int_{-\infty}^{\infty} \Phi(x + \Delta_H)^{L-1}\phi(x - r_{HM} + \Delta_H)\, dx \qquad \text{(change of variables)} \\
&= \int_{-\infty}^{\infty} \Phi(x + \Delta_H)^{L-1}\phi(x - r_{HH})\, dx \qquad \text{(definition of } \Delta_H\text{)} \\
&\geq \int_{-\infty}^{\infty} \Phi(x)^{L-1}\phi(x - r_{HH})\, dx \qquad (\Delta_H > 0 \text{ and } \Phi(\cdot) \text{ is increasing}) \\
&= A_{HH}
\end{aligned}
$$

□

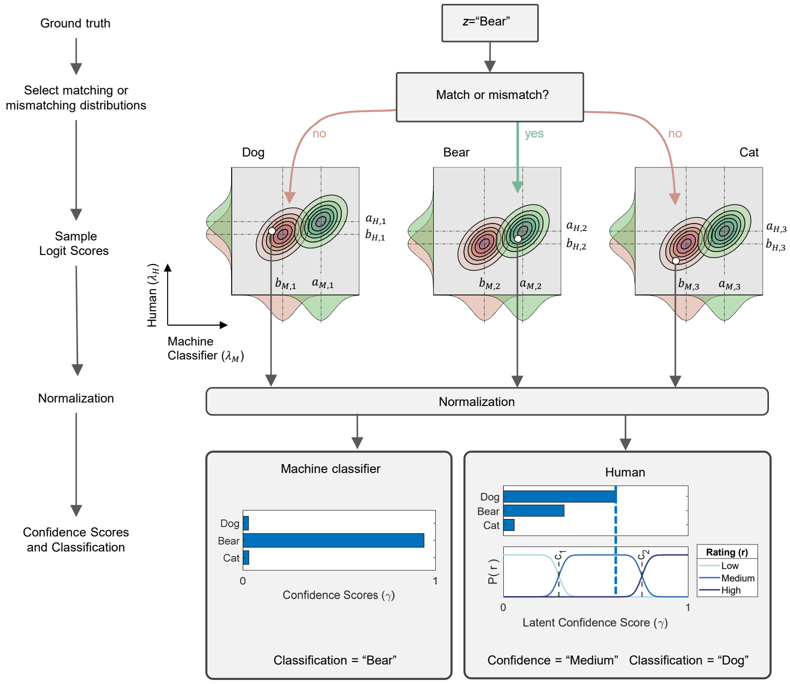

Fig. S1. Illustration of the generative process of the Bayesian model that produces the classification and confidence scores for a single human (H) and machine classifier (M). In the example, there are three classes and the ground truth (z) for a particular image is "Bear". The ground truth selects for each label a bivariate normal distribution with means (a_M, a_H) shown in green or (b_M, b_H) shown in red when the ground truth matches or mismatches the label respectively. A single sample (white circle) is taken from each selected bivariate normal distribution to produce the correlated logit scores (λ) for the human and machine classifier. The separation of the means between matching and mismatching distribution $(a - b)$ determines the discrimination ability of the classifier for that class whereas the mean of the mismatching distribution (b) determines response bias for that class. In this example, the human classifier has a response bias for "Dog". For the machine classifier, the logit scores are transformed to observable probabilities (γ). For the human, a softmax is applied to the latent confidence scores (γ) to determine the classification (dog) and an ordinal probit model is used to sample the observed confidence rating ("Medium").

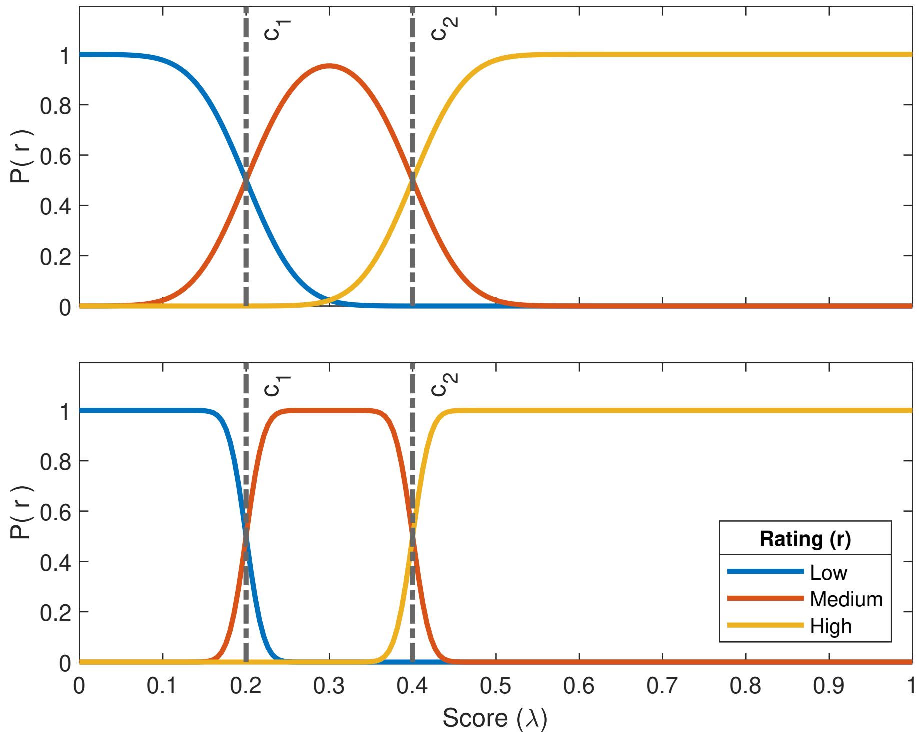

Fig. S2. Illustration of the ordered probit model for three ratings. Top and bottom panels are produced with $\delta = 20$ and $\delta = 60$ respectively

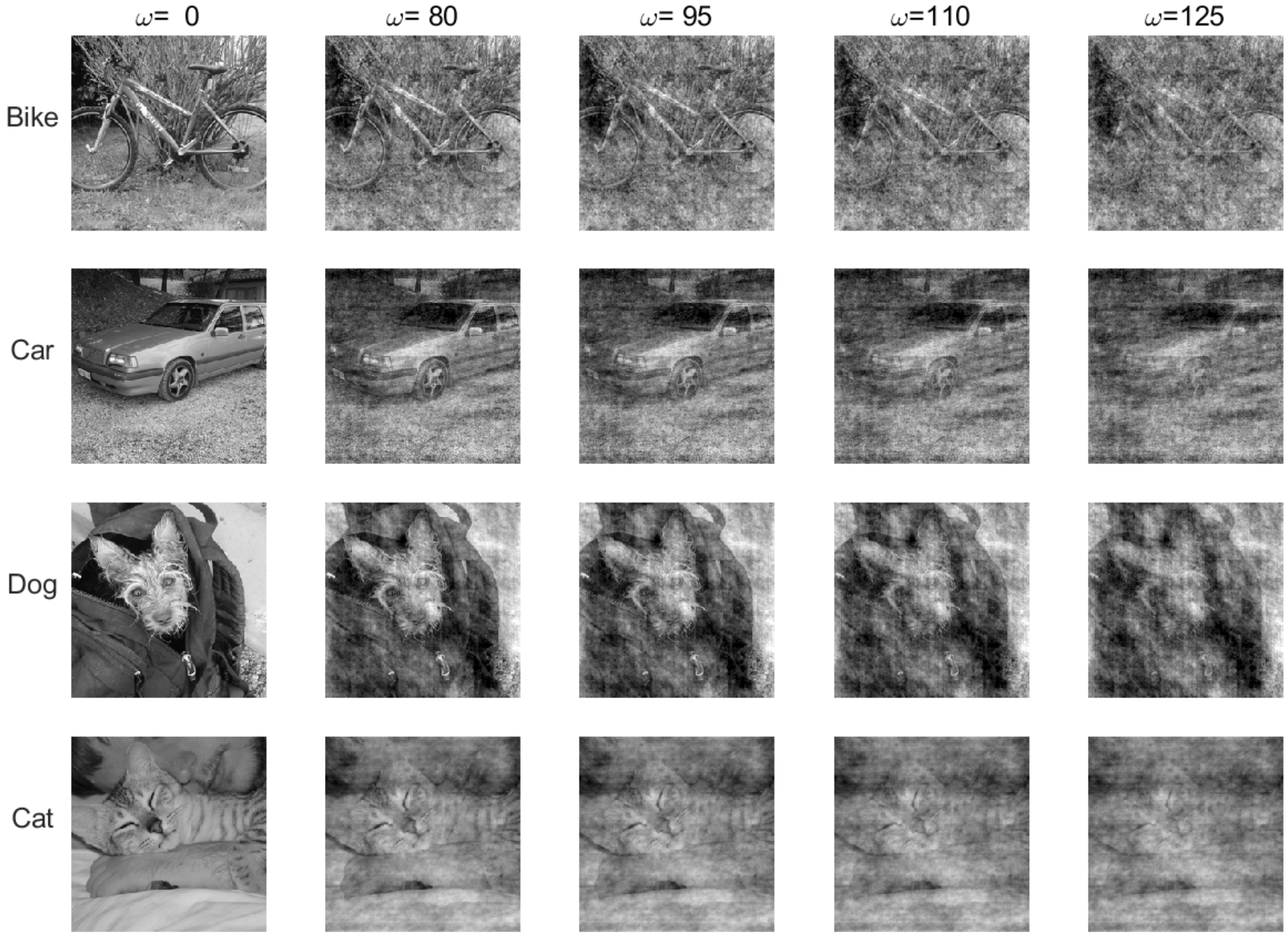

Fig. S3. Examples of images from different categories without phase noise (leftmost column) and the four noise levels used in the image classification experiments (ω=80, 95, 110, and 125.)

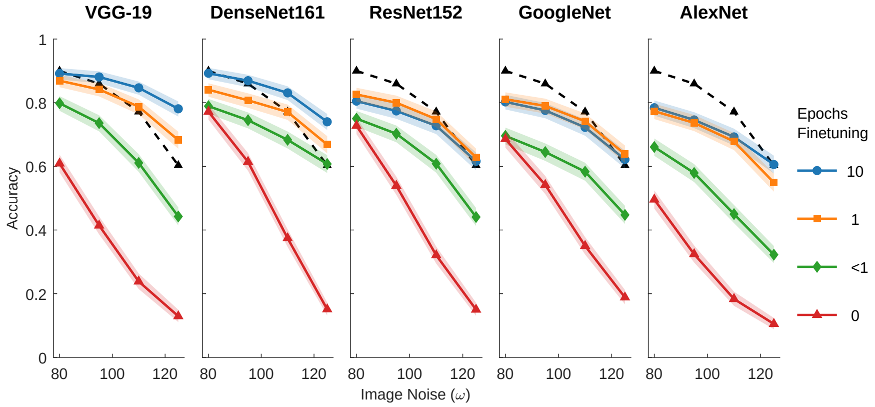

Fig. S4. Classification performance of individual humans (dashed line) and five different machine classifiers as a function of image noise. For the machine classifiers, performance is shown across levels of fine-tuning. Human performance is replicated across panels to facilitate visual comparison. Error bars reflect 95% confidence intervals of the mean based on a binomial model.

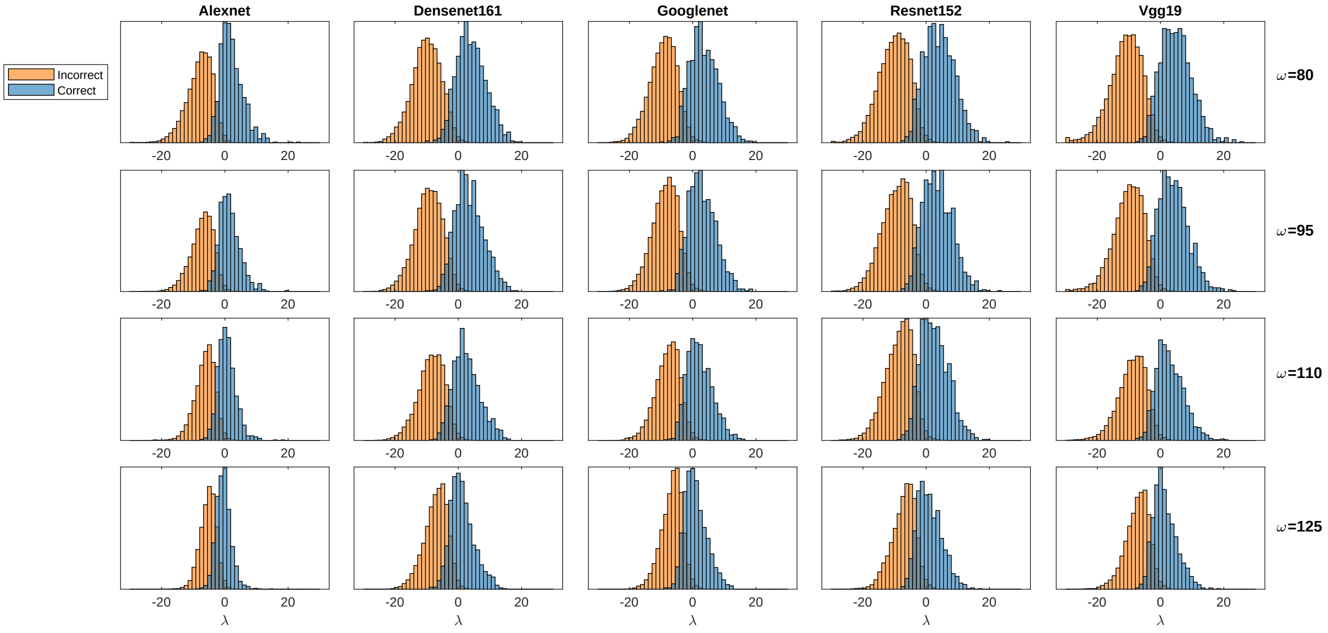

Fig. S5. Distributions of λ logit scores for correct and incorrect classes. Results are separated by machine classifiers (columns) and levels of image noise ω (rows). The machine classifiers were fine-tuned for 1 epoch.

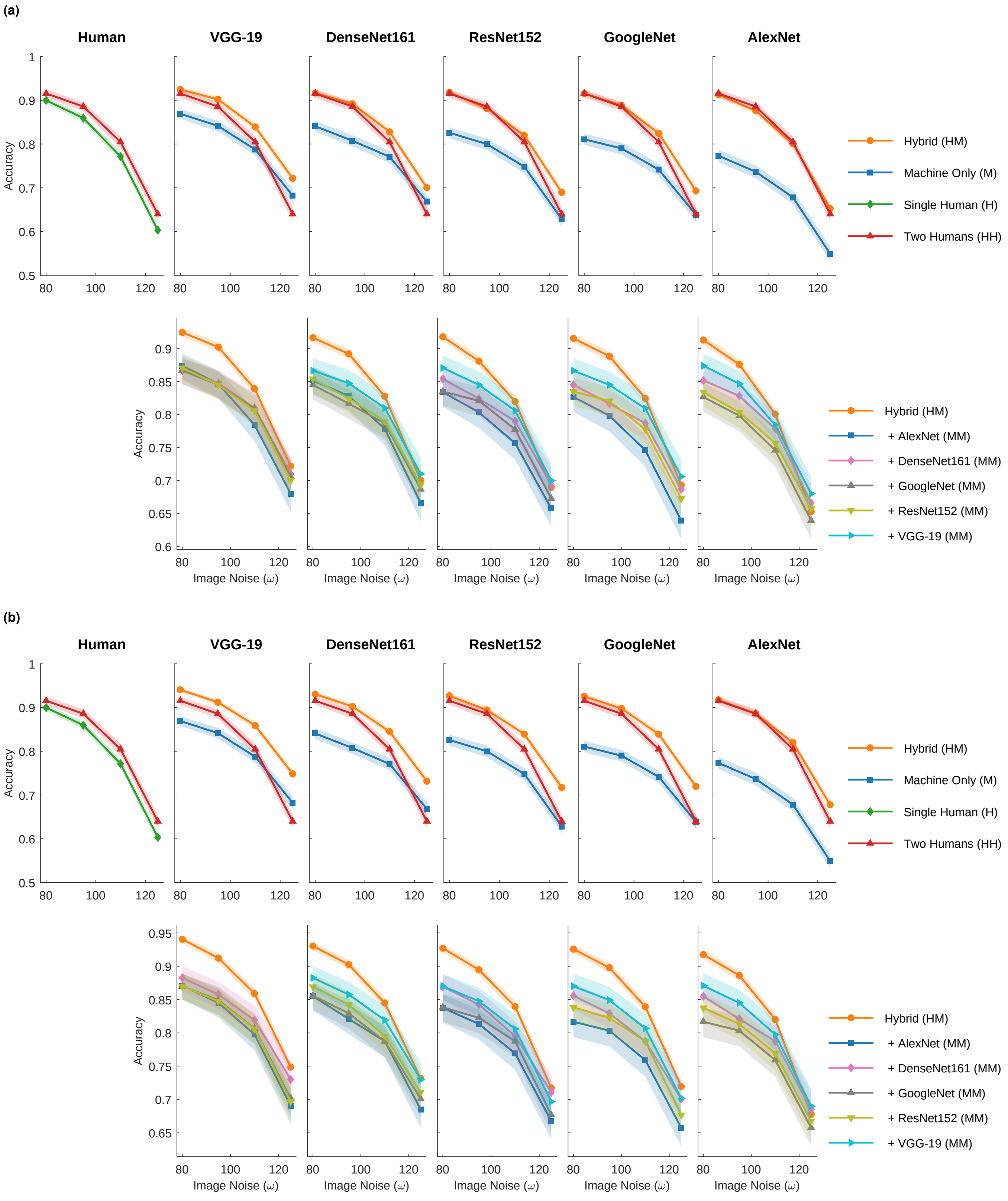

Fig. S6. Accuracy results for the Bayesian combination model with machine classifiers scores that are partially observed and discretized (a), fully observed and continuous (b). Results are shown as a function of image noise (horizontal axis) and classifier (columns). Error bars reflect 95% confidence interval of the mean based on a binomial model. Machine classifiers are finetuned for 1 epoch.

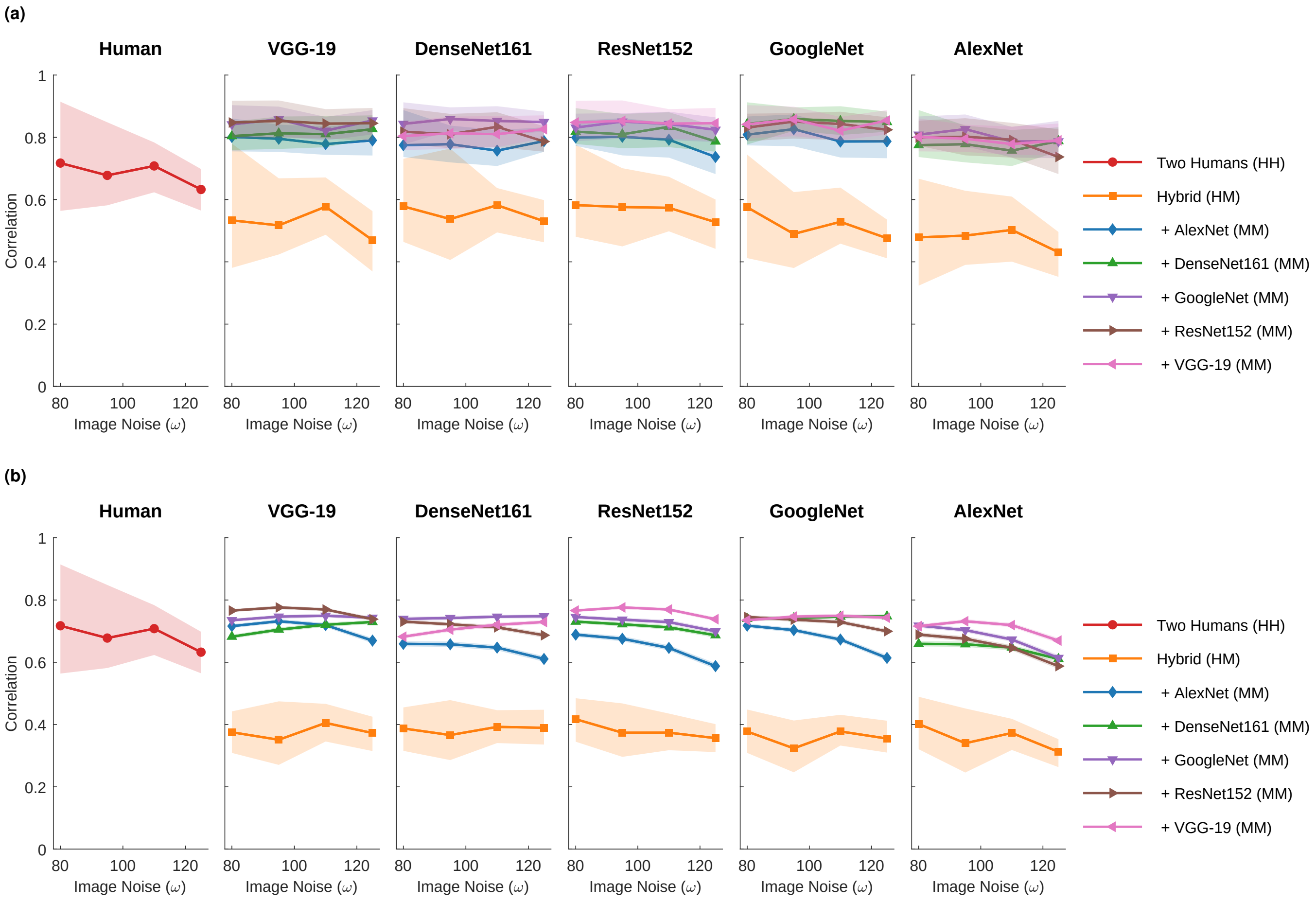

Fig. S7. Posterior distributions of the latent correlation in the Bayesian combination model with machine classifiers scores that partially observed and discretized (a), fully observed and continuous (b). Colored areas reflect 95% credible intervals. Machine classifiers are finetuned for 1 epoch.

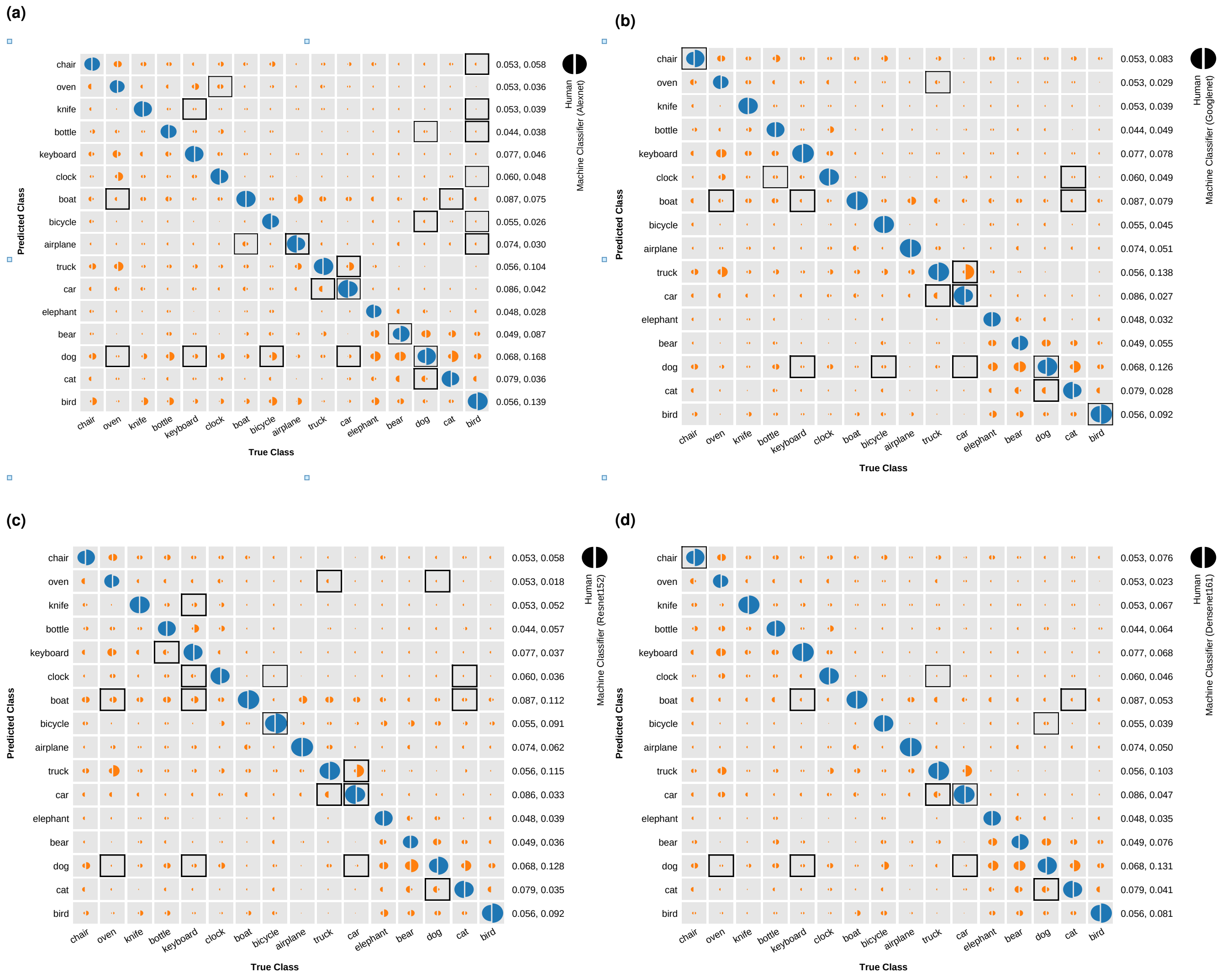

Fig. S8. Confusion matrix for human (left-half circles) and machine classifier (right half circles) predictions for four types of machine classifiers (panels a-d: Alexnet, Googlenet, Resnet152, and Densenet161). Circle sizes represent the proportion of correct (blue) and error (orange) responses. Numbers on right side indicate the overall proportion of predicted class labels for the human (left) and machine classifier (right). Highlighted boxes have significantly different proportions as assessed by a Bayes factor test for the difference between two binomials ($BF > 10$, thin black box; $BF > 100$, thick black box). The data is based on the most challenging image noise (ω=125) condition in the classification experiment. The machine classifiers were fine-tuned for 1 epoch.

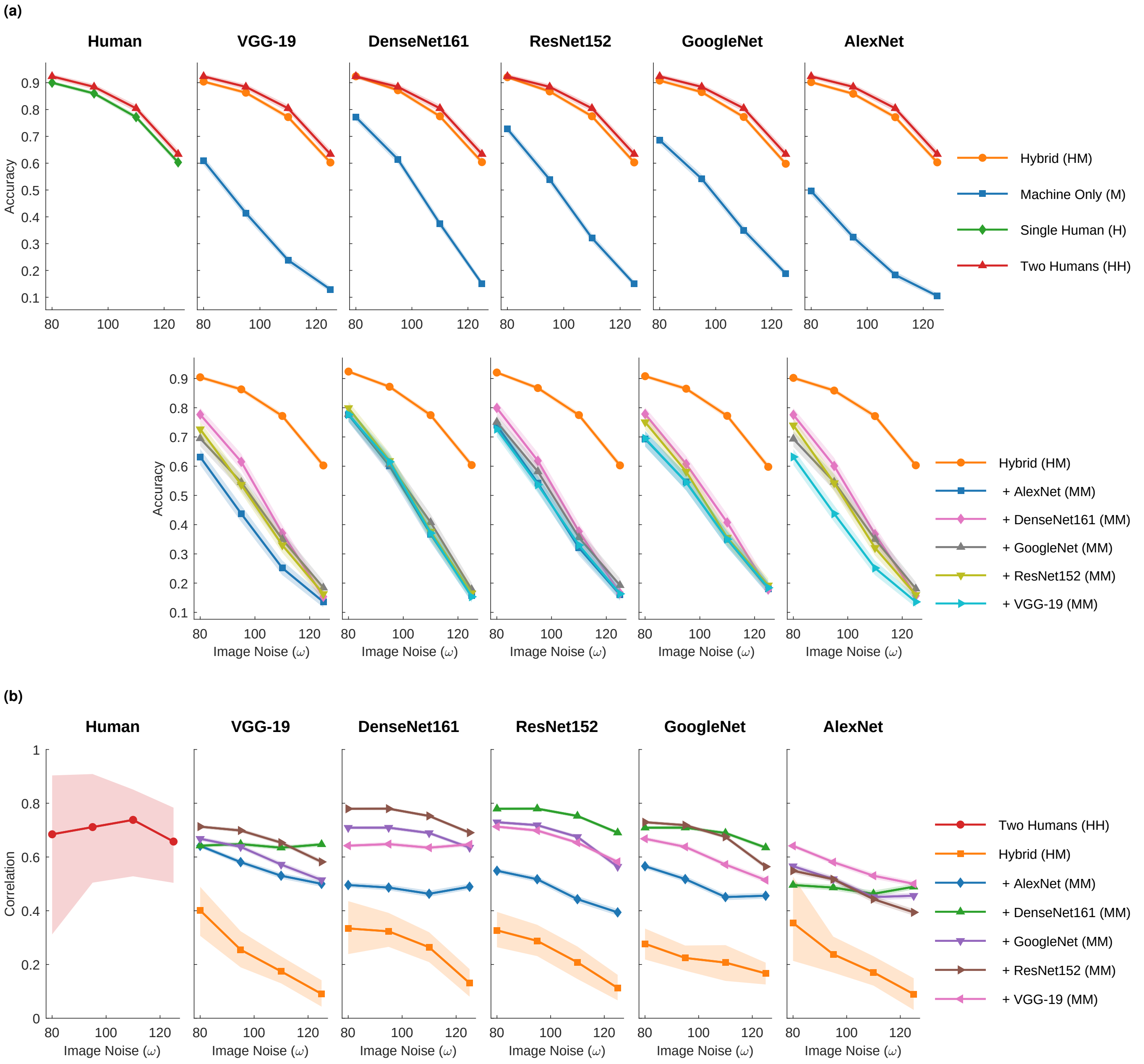

Fig. S9. Accuracy (a) and correlation (b) results for the Bayesian combination model with machine classifiers finetuned for 0 epochs (baseline). Results are shown as a function of image noise (horizontal axis) and classifier (columns). Error bars reflect 95% confidence interval of the mean based on a binomial model.

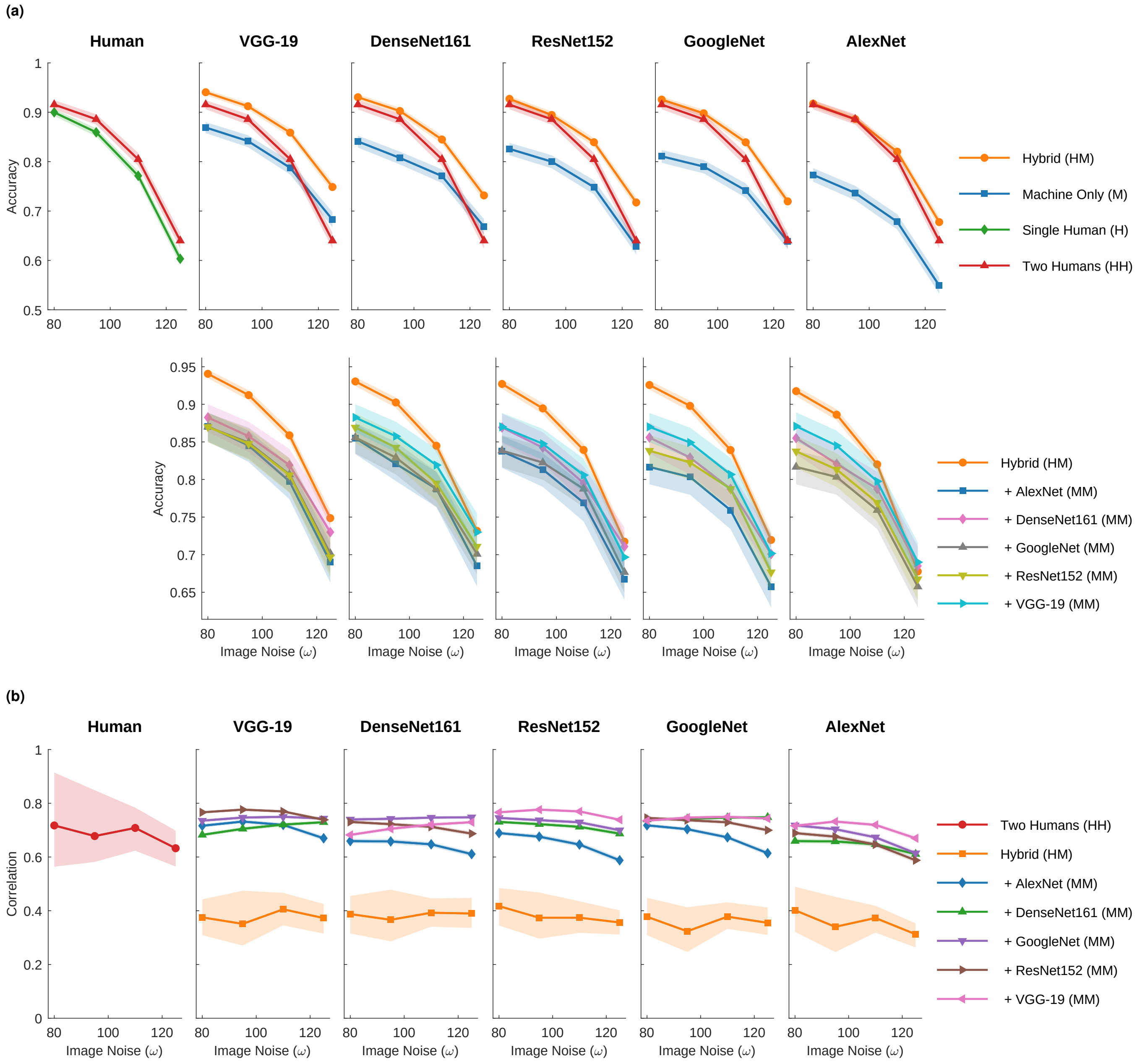

Fig. S10. Accuracy (a) and correlation (b) results for the Bayesian combination model with machine classifiers finetuned for 1 epoch. Results are shown as a function of image noise (horizontal axis) and classifier (columns). Error bars reflect 95% confidence interval of the mean based on a binomial model.

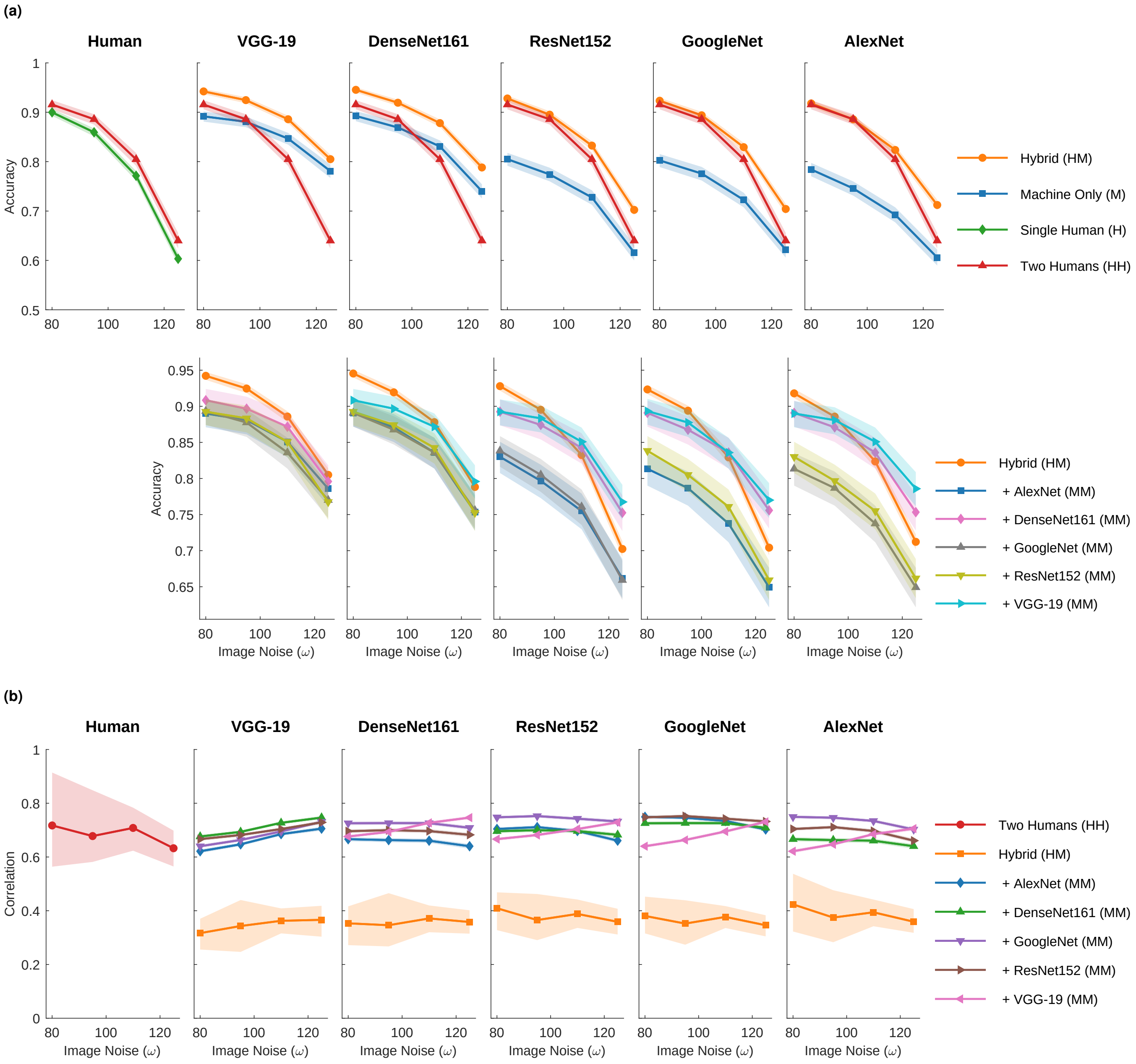

Fig. S11. Accuracy (a) and correlation (b) results for the Bayesian combination model with machine classifiers finetuned for 10 epochs. Results are shown as a function of image noise (horizontal axis) and classifier (columns). Error bars reflect 95% confidence interval of the mean based on a binomial model.

Table S1. Posterior means of discrimination (d) and mean-centered response bias (b) parameters for the human (H) and the VGG-19 machine classifier (M) for two levels of image noise (ω). The relative discrimination advantage of human participants over the machine classifier ($d_H - d_M$) is visualized with color bars. Results are based on the Bayesian combination model with the VGG-19 machine-classifier fine-tuned for 1 epoch and both human and machine confidence scores are used.

	$\omega = 110$					$\omega = 125$				
Class	b_H	b_M	d_H	d_M	$d_H - d_M$	b_H	b_M	d_H	d_M	$d_H - d_M$
chair	$+0.15$	-0.18	2.41	2.32	$+0.09$	$+0.11$	-0.27	1.77	2.04	-0.27
oven	$+0.26$	-0.01	2.05	2.26	-0.21	$+0.07$	$+0.09$	1.73	1.97	-0.25
knife	-0.52	-1.19	3.96	2.85	$+1.11$	-0.41	-1.19	3.19	2.54	$+0.65$
bottle	-0.24	$+1.11$	2.89	2.51	$+0.37$	-0.40	$+1.01$	2.45	2.19	$+0.26$
keyboard	$+0.29$	-0.98	3.05	2.76	$+0.29$	$+0.23$	-0.95	2.34	2.39	-0.05
clock	-0.13	-0.47	3.58	2.79	$+0.80$	-0.23	-0.54	3.25	2.49	$+0.77$
boat	$+0.43$	$+0.68$	2.44	3.05	-0.61	$+0.46$	$+0.57$	1.73	2.72	-0.99
bicycle	-0.59	-0.88	3.97	3.29	$+0.68$	-0.36	-0.75	2.95	2.86	$+0.09$
airplane	$+0.08$	-1.63	3.65	3.35	$+0.30$	$+0.11$	-1.59	3.02	3.06	-0.04
truck	-0.02	$+1.64$	3.04	2.74	$+0.30$	-0.11	$+1.46$	2.39	2.48	-0.09
car	$+0.33$	-0.77	3.51	2.13	$+1.37$	$+0.26$	-0.78	2.98	1.94	$+1.03$
elephant	-0.16	-1.12	3.13	2.86	$+0.26$	-0.16	-0.91	2.09	2.29	-0.20
bear	-0.29	-0.07	2.44	2.18	$+0.27$	$+0.05$	$+0.13$	1.49	1.81	-0.32
dog	$+0.32$	$+2.09$	2.23	2.50	-0.27	$+0.28$	$+1.98$	1.46	2.11	-0.66
cat	$+0.15$	$+0.34$	2.97	2.66	$+0.31$	$+0.24$	$+0.46$	2.25	2.16	$+0.09$
bird	-0.07	$+1.44$	2.94	3.44	-0.50	-0.15	$+1.27$	2.26	3.01	-0.74

Table S2. Accuracy for human-machine classifier combinations across image noise and different types of combination models that vary the presence or absence of an error model, human confidence scores, and machine classifier confidence scores. The results are separated by the 5 machine classifiers. Each accuracy result is based on 7,200 observation.

					Image Noise (ω)			
Machine Classifier	#	Error Model	Human Confidence	Machine Confidence	80	95	110	125
AlexNet								
	1	✓	✓	✓	0.917	0.889	0.828	0.712
	2	✗	✓	✓	0.915	0.886	0.821	0.676
	3	✓	✗	✓	0.911	0.884	0.819	0.701
	4	✗	✗	✓	0.914	0.881	0.809	0.662
	5	✓	✓	✗	0.900	0.869	0.804	0.654
	6	✗	✓	✗	0.897	0.866	0.799	0.651
	7	✓	✗	✗	0.887	0.853	0.779	0.628
	8	✗	✗	✗	0.896	0.861	0.766	0.588
DenseNet161								
	1	✓	✓	✓	0.934	0.907	0.853	0.755
	2	✗	✓	✓	0.929	0.902	0.843	0.733
	3	✓	✗	✓	0.932	0.902	0.851	0.744
	4	✗	✗	✓	0.926	0.898	0.836	0.721
	5	✓	✓	✗	0.915	0.885	0.831	0.710
	6	✗	✓	✗	0.906	0.876	0.819	0.691
	7	✓	✗	✗	0.904	0.874	0.812	0.689
	8	✗	✗	✗	0.894	0.859	0.771	0.663
GoogleNet								
	1	✓	✓	✓	0.934	0.913	0.852	0.749
	2	✗	✓	✓	0.925	0.901	0.841	0.726
	3	✓	✗	✓	0.930	0.908	0.845	0.739
	4	✗	✗	✓	0.922	0.895	0.828	0.706
	5	✓	✓	✗	0.910	0.887	0.826	0.710
	6	✗	✓	✗	0.900	0.881	0.818	0.698
	7	✓	✗	✗	0.902	0.882	0.815	0.679
	8	✗	✗	✗	0.897	0.857	0.762	0.634
ResNet152								
	1	✓	✓	✓	0.934	0.903	0.848	0.745
	2	✗	✓	✓	0.927	0.894	0.839	0.722
	3	✓	✗	✓	0.929	0.900	0.841	0.736
	4	✗	✗	✓	0.927	0.894	0.825	0.708
	5	✓	✓	✗	0.906	0.875	0.815	0.694
	6	✗	✓	✗	0.900	0.867	0.811	0.679
	7	✓	✗	✗	0.899	0.865	0.794	0.663
	8	✗	✗	✗	0.893	0.859	0.762	0.618
VGG-19								
	1	✓	✓	✓	0.944	0.921	0.868	0.779
	2	✗	✓	✓	0.940	0.913	0.858	0.754
	3	✓	✗	✓	0.939	0.916	0.863	0.771
	4	✗	✗	✓	0.936	0.908	0.852	0.738
	5	✓	✓	✗	0.923	0.900	0.840	0.735
	6	✗	✓	✗	0.914	0.890	0.828	0.711
	7	✓	✗	✗	0.912	0.887	0.828	0.711
	8	✗	✗	✗	0.893	0.856	0.785	0.676

References

1. O Russakovsky, et al., Imagenet large scale visual recognition challenge. *Int. J. Comput. Vis.* **115**, 211–252 (2015).
2. R Geirhos, et al., Generalisation in humans and deep neural networks in *Thirty-second Annual Conference on Neural Information Processing Systems (NeurIPS 2018).* (Curran), pp. 7549–7561 (2019).
3. FA Wichmann, DI Braun, KR Gegenfurtner, Phase noise and the classification of natural images. *Vis. Res.* **46**, 1520–1529 (2006).
4. A Krizhevsky, I Sutskever, GE Hinton, Imagenet classification with deep convolutional neural networks. *Adv. Neural Inf. Process. Syst.* **25**, 1097–1105 (2012).
5. G Huang, Z Liu, L Van Der Maaten, KQ Weinberger, Densely connected convolutional networks in *Proceedings of the IEEE Conference on Computer Vision and Pattern Recognition.* pp. 4700–4708 (2017).
6. C Szegedy, et al., Going deeper with convolutions in *Proceedings of the IEEE Conference on Computer Vision and Pattern Recognition.* pp. 1–9 (2015).
7. K He, X Zhang, S Ren, J Sun, Deep residual learning for image recognition in *Proceedings of the IEEE Conference on Computer Vision and Pattern Recognition.* pp. 770–778 (2016).
8. K Simonyan, A Zisserman, Very deep convolutional networks for large-scale image recognition in *International Conference on Learning Representations.* (2015).
9. M Plummer, , et al., JAGS: A program for analysis of Bayesian graphical models using Gibbs sampling. (year?).
10. NA Macmillan, CD Creelman, *Detection theory: A user's guide.* (Psychology Press), (2004).
